# The Kinetics of Polymer Adsorption in Transport Through Porous Media: Basic Theory

**DOI:** 10.3390/polym18192394

**Published:** 2026-09-30

**Authors:** Kenneth S. Sorbie, Randall S. Seright, Alan Beteta, Dongmei Wang, Celia Melo da Silva

**Affiliations:** 1 Heriot-Watt University, Edinburgh EH14 4AS, UK; a.beteta@hw.ac.uk (A.B.); csilva@snf.com (C.M.d.S.); 2 New Mexico Institute of Mining and Technology, Socorro, NM 87801, USA; randy.seright@nmt.edu; 3 University of North Dakota, Grand Forks, ND 58202, USA; 4 SNF Floerger, 42160 Andrézieux-Bouthéon, France

**Keywords:** polymer adsorption, kinetics of polymer adsorption, polymer transport through porous media, transport equation with kinetic polymer adsorption

## Abstract

A polymer transport and kinetic adsorption model is developed and applied to assist in the understanding of some recent results in polymer flow through porous media. The coupled equations in dimensionless form show that the system is governed by 3 dimensionless numbers, 2 of which are important: the Adsorption number (N_Ad_) and the adsorption Damköhler number (N_Da-A_). This leads to a wide range of behaviours in terms of the polymer effluent profiles, which are in broad qualitative agreement with experimental observations. The hypothesis is that it is the kinetics of the system (principally N_Da-A_) that is strongly controlling the experimental observations. The model also predicts that the way to establish if this is true is to conduct a polymer core flood including “shut-in” stages where flow is stopped, then resumed after some period of time, depending on the kinetic adsorption timescale. Preliminary experiments support this, and a following paper will present these experimental results directly simulated using our model.

## 1. Introduction

### 1.1. Background on Polymer Adsorption—Equilibrium and Kinetics

**Figure 1:**
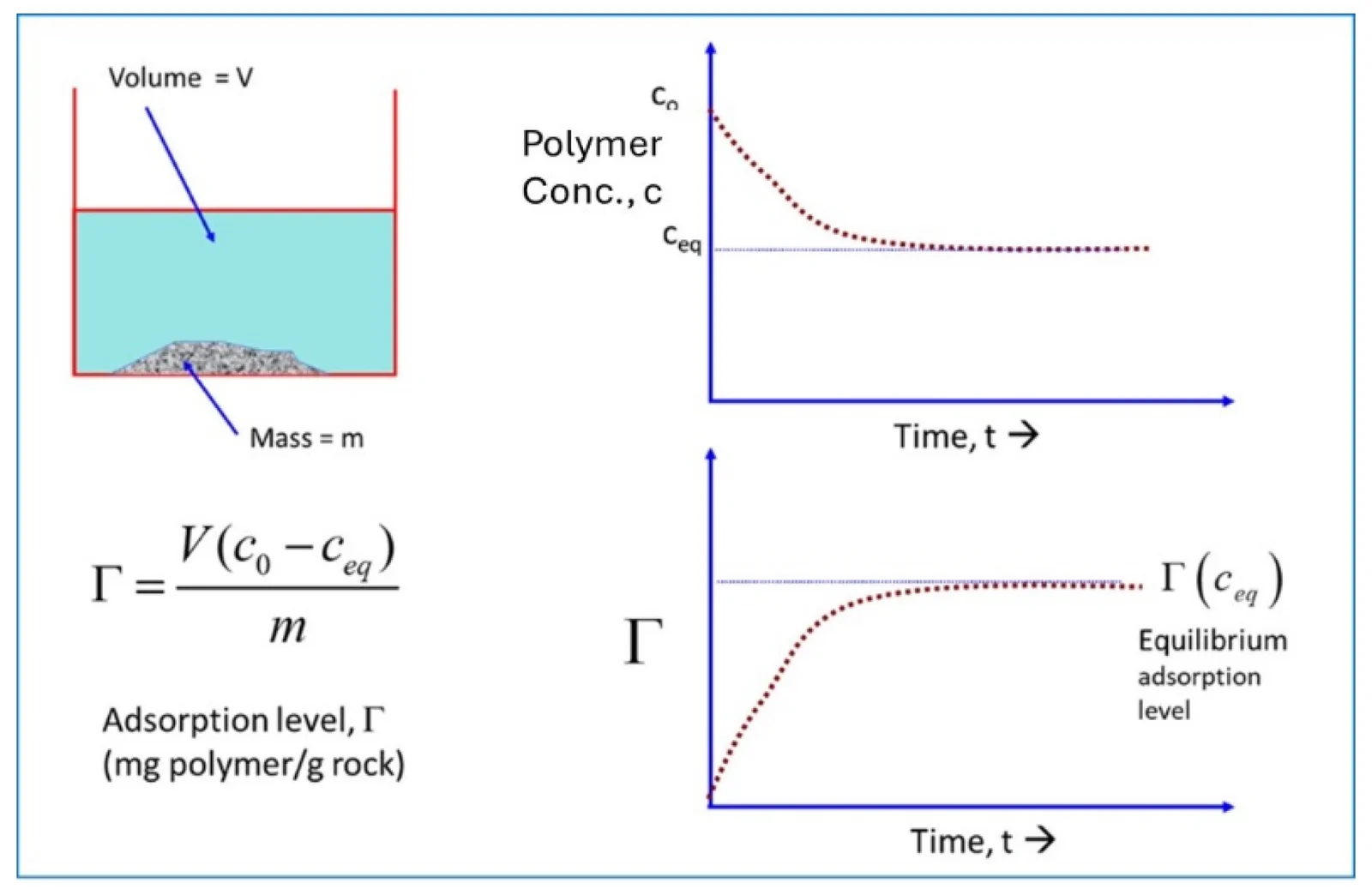
Schematic of a bulk (beaker) static adsorption measurement of polymer adsorption on a mineral substrate (e.g., quartz sand, clay mineral, calcite, etc.).

Polymer adsorption onto a solid substrate is important in a number of industrial processes such as water treatment, mineral processing and enhanced oil recovery (EOR) [1,2,3,4,5]. Figure 1 shows a schematic outline of a bulk polymer adsorption measurement for a polymer solution of initial (t = 0) concentration, c_o_ (mg/L); this is an almost exact schematic of the actual polymer/mineral adsorption experiment. The polymer solution volume is V (in L), and the mass of substrate is m (g). As the polymer adsorbs on the solid substrate, the corresponding polymer solution concentration declines until, after sufficient time, it reaches an equilibrium value, denoted c_eq_ in Figure 1. By simple material balance, this corresponds to an equilibrium polymer adsorption level, Γ (mg/g) given by Equation (1), as follows [1]:

(1) $$\Gamma \left(c_{\mathrm{eq}}\right)=\frac{V(c_{0}-c_{\mathrm{eq}})}{m}$$

A single experiment of this type will, for a given well-defined system*, give a single point, Γ *vs*. *c_eq_* on the polymer/substrate equilibrium adsorption isotherm; *i.e., at a given set of conditions—temperature (T), solution composition, pH, specific mineral, specific polymer, etc. By carrying out this process for a number of initial polymer concentrations, then a series of points on the equilibrium adsorption isotherm can be built up. In most cases, it is possible to fit one of the common analytical forms of the adsorption isotherm to this data, e.g., a Langmuir or Freundlich form. A large number of possible alternative analytical forms of the adsorption isotherm are summarised in recent reviews [6,7]. Many of the analytical adsorption isotherms have some theoretical basis, often based on the assumed kinetics of how the equilibrium is reached, and this is the case for both Langmuir and Freundlich isotherms, as discussed below. However, the various analytical forms of adsorption isotherms can also be viewed and utilised simply as empirical forms that fit the polymer/substrate equilibrium adsorption data [6,7]. From the perspective of data fitting, more flexible isotherm forms may be used, such as the extended Langmuir [8] and the hybrid Langmuir/Freundlich model [9].

Any polymer/solvent/substrate system will come to equilibrium at some time, and such experiments can be used to construct the equilibrium adsorption isotherm, Γ(c), which is a function of polymer concentration, c. Furthermore, for many polymer adsorption applications, the *kinetics* of the polymer/substrate adsorption process can be very important; i.e., how long it takes for equilibrium adsorption to be reached. The literature on polymer adsorption for many applications is vast, and here we will consider mainly the ***kinetics*** of the adsorption process, with a special focus on adsorption within porous media, i.e., during flow through sand/mineral packs or rock cores.

Whether in bulk solution or in a porous medium, the adsorption process for polymers at the mineral surface is considerably more complex than that for the adsorption of small molecular species [1,2,4,10]. For a high molecular weight flexible chain molecule in aqueous solution (e.g., hydrolysed polyacrylamide (HPAM) in water), adsorption at the surface of any substrate involves several stages, as follows:

(i) *Diffusion to the surface*: polymer transport to the adsorbing surface by diffusion of the high molecular weight polymer molecule is relatively “slow” compared with smaller molecular species, since D_0_ ~ 1 × 10^−12^ to 1 × 10^−13^ m^2^/s (D_0_ is the molecular diffusion coefficient);
(ii) *Molecular anchoring:* surface contact and initial attachment of the macromolecule at one or two polymer segments giving an initial “anchoring” of the polymer chain, with the remainder of the flexible chain being in the solvent;
(iii) *Polymer chain surface conformation:* several adjustments in chain conformation then take place, including the attachment of further chain segments to the surface (known as “trains”), which form “loops” of the now bound polymer chain in the solvent and “tails” of polymer at each end of the polymer chain, also in the solution. This is described by statistical mechanical lattice theory, which has an extensive literature [2,11,12,13];
(iv) *Additional polymer surface “crowding”:* simultaneously, as more polymer molecules arrive on the surface, a “crowding” effect occurs, and the already adsorbed molecules may adjust their configuration by forming larger solution loops, which has the physical effect of extending the adsorbed polymer layer thickness; and
(v) *Relaxation to equilibrium:* the gradual relaxation of the flexible chain polymer molecule and its neighbours on the surface occurs, thus reaching a (dynamic) equilibrium surface conformation of adsorbed polymer loops, trains and tails.

The thermodynamic driving force for the polymer-surface adsorption process is the energy of attachment of the (many) polymer trains on the surface (usually by displacing surface-bound solvent molecules), which must counterbalance the loss of conformational entropy of the free long chain in solution. A further complication arises in the adsorption of polymers due to their polydispersity, i.e., the wide molecular weight distribution of the polymer [14]. The smaller molecules with much higher diffusion coefficients adsorb much faster than the higher molecular weight components; however, these larger species will gradually displace the lower molecular weight species, which again may not be a rapid process. A feature of this entire adsorption process is that it may take from **hours to several days** to reach full equilibrium of the system even in a well-mixed system; this is well described in references [2,4]. Even in their studies of HPAM adsorption on Millipore filters, Gramain and Myard [10] found that it took 3 or 4 days for the adsorption process to come to equilibrium.

In light of the discussion above, we must consider whether the kinetic measurements of polymer adsorption rates made in bulk static adsorption tests carried out in a beaker, as shown in Figure 1, are relevant to the adsorption process in porous media. For this beaker-scale case, diffusion from the bulk liquid to the solid surface within the granular solid mass may require a substantially longer time compared to that for liquid within a porous medium. Within a pack or rock core, the maximum distance for diffusion will generally be less than 50 µm. A flexible coil polymer, such as HPAM, of about 20 MDa with a D_o_ between 10^−12^ m^2^/s and 10^−13^ m^2^/s would take between ~0.17 h and ~ 1.7 to travel a distance l = 50 μm, according to the Einstein-Schmoluchowski formula, t = l^2^/(2dD_0_) (d is the dimension, assumed 2). (In contrast, a typical small molecule in solution would take ~0.3s to travel an average displacement of 50μm) And this arrival time might be increased if the polymer solution itself were concentrated (by polymer molecular entanglement) and viscous effects (which reduce D_o_). However, this is not the longest time in the polymer adsorption process; stages (ii)–(iv) will generally take much longer than this [4].

Recent work [15,16] conducted using the method illustrated in Figure 1 has indicated that for certain water-soluble polymers, such as high molecular weight partially hydrolysed polyacrylamide (HPAM), the time scale to reach adsorption equilibrium may, under some circumstances, be several days. In this experimental work, the system was occasionally stirred to assist the adsorption process, but the caution expressed above should be kept in mind, since we might expect such timescales to be lower within porous media. This has an obvious effect on exactly how long a time period the adsorption experiment should be carried out if the correct equilibrium adsorption is to be achieved.

### 1.2. Dynamic Polymer Adsorption in Porous Media

For cases of polymer flow through porous media, a longer timescale to reach equilibrium adsorption also has implications on how polymer core floods are carried out, in particular on the chosen volumetric flow rate, q; more precisely, we show that it is the fluid pore velocity, ν = q/(Aϕ) which should be considered (Where q is in units such as cm^3^/h, A is the cross-sectional area (cm^2^) of the flow pack or core, and φ is the porosity. Pore velocity, ν, would then be in cm/h. Note that some papers in the literature use the related Darcy velocity, u = q/A). If polymer adsorption onto a porous medium, such as a sandstone rock or a sand pack, is being measured, then the *residence time* in this experiment will be relevant since it may be too short to reach equilibrium. The fluid residence time, T_r_, is given by T_r_ = V_p_/q, where V_p_ is the core pore volume. If equilibrium is assumed (incorrectly) in such a system when making the adsorption estimates, then erroneous results will be obtained.

We now consider the relevant literature on the adsorption of polymers in dynamic (flowing) mineral pack and core floods. To measure the level of adsorption (Γ_max_) in such a system, the method is quite different from the bulk adsorption beaker test illustrated in Figure 1, as discussed below in the context of an actual example.

Many dynamic core and pack flooding polymer adsorption experiments have been presented in the literature, and an early review of these has been presented [1]. However, some of the more recent results find polymer production profiles that show a puzzling type of “tailing” in the observed polymer effluents [17,18,19]. The cause of this effluent behaviour has been uncertain in the interpretation of these experiments, and it has been attributed to various effects such as bridging adsorption [18] and a dual time scale for equilibrium adsorption onto quartz versus clays [19]. Indeed, kinetic effects have also been suspected in the interpretation of this work.

**Figure 2:**
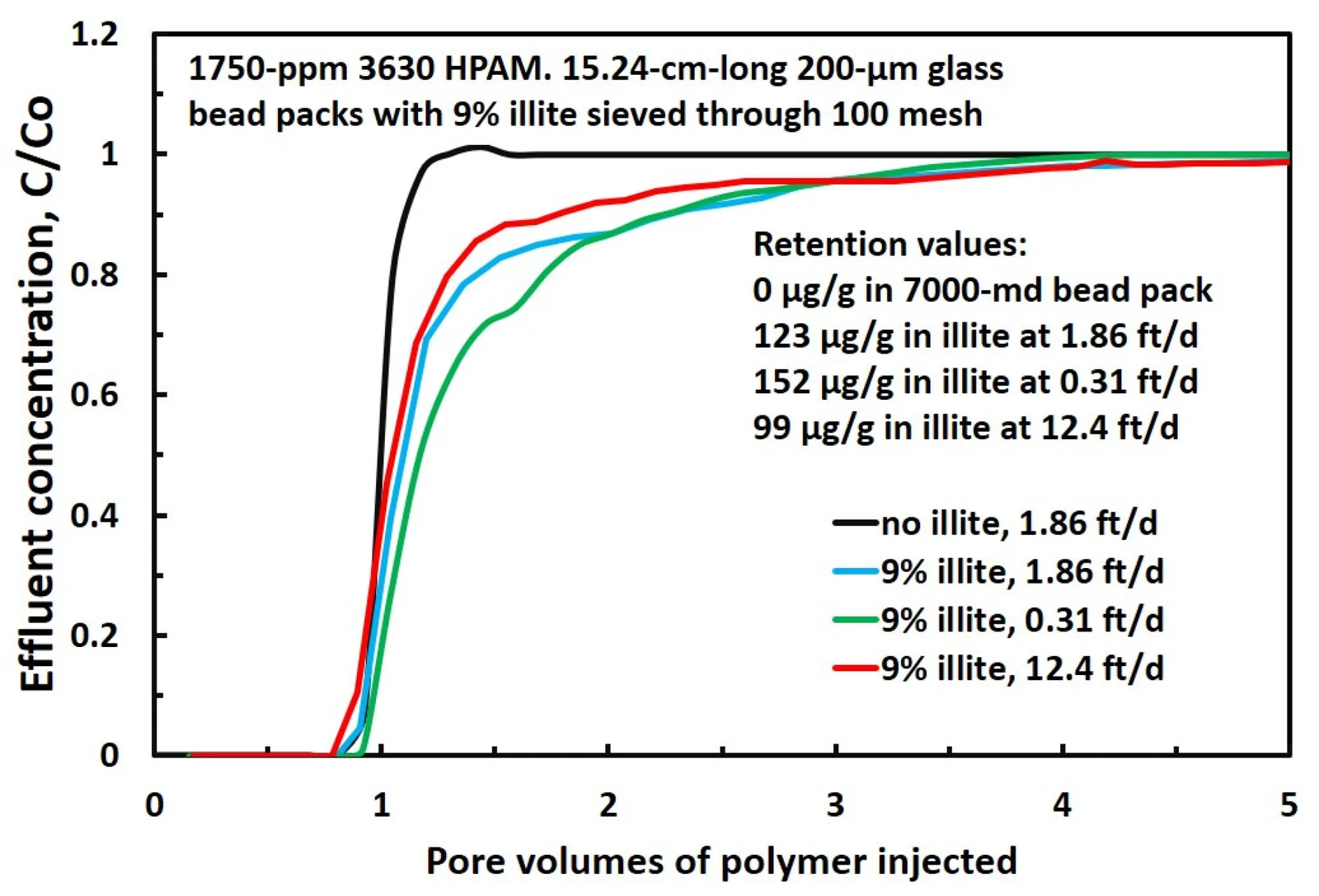
Polymer concentration effluent values vs pore volume (PV) injected showing the influence of injection rate on the “tailing” effect; note that the velocity referred to in this figure and in Figure 3 is the Darcy velocity, u, where u =q/A (see text).

**Figure 3:**
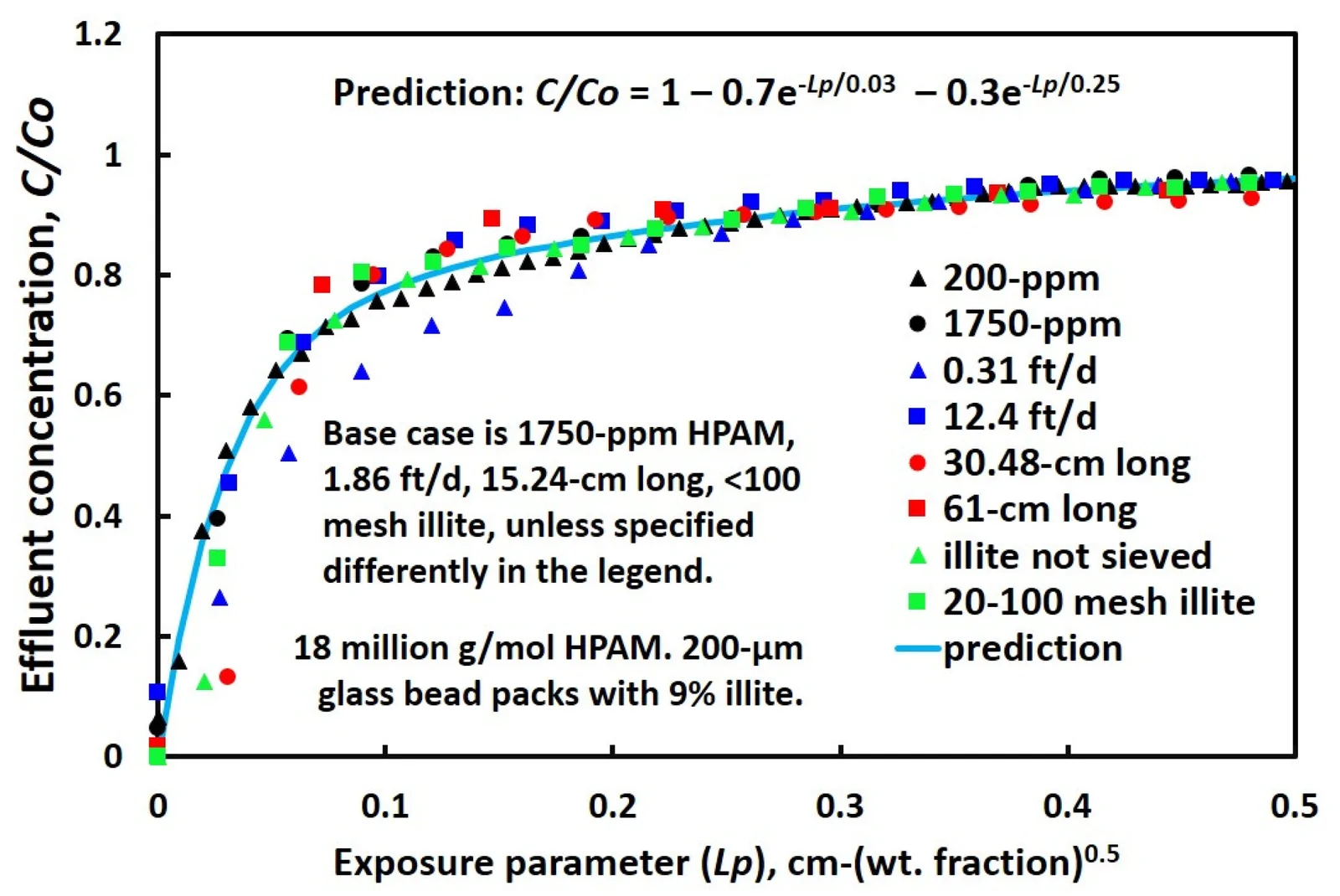
Dimensionless effluent polymer concentration (c/c_0_) vs. the Exposure parameter (Equation (2)) showing the influence of various parameters on the “tailing” effect; velocities refer to the Darcy velocity, u =q/A (see text).

During extensive dynamic-retention (i.e., coreflood or mineral pack) experiments associated with the Milne Point polymer flood, Seright and Wang [18,19] observed this “tailing phenomenon” when the clays, illite or kaolinite, were present in quartz-based sands. Figure 2 and Figure 3 show this tailing behaviour as a function of injection rate and other conditions for an HPAM polymer of Mw = 18-million-g/mol (MDa) in glass bead packs containing 9% added illite. Figure 2 and Figure 3 are replotted from the data in Seright and Wang [18].

In Figure 3, the dimensionless effluent concentration of polymer (c/c_0_) shows a good correlation with the “exposure parameter”, *L_p_*, which is given by Equation (2) [19]:

*L_p_ = (t − t_b_)u C*^−0.5^ (2)

where *t* is total time since the start of polymer injection, *t_b_* is time at polymer arrival at the end of the core, *u* is superficial or Darcy velocity, and *C* is polymer concentration. To account for the tailing phenomenon in the above two figures, Seright and Wang [19] speculated that there was a different (slower) time or “exposure” constant for polymer adsorption onto illite versus onto quartz or glass. The timescale for polymer injection ranged from 2.7 h (for the 12.4-ft/d experiment) to 108 h (for the 0.31-ft/d experiment). These flow velocities (as Darcy velocities, u = q/A) correspond to residence times of ~0.19 h (12.4 ft/day) and ~7.7 h (0.31 ft/day).

As is the general practice in polymer core flooding, the experimental results in Figure 2 are used to measure the level of polymer adsorption in the core. The adsorption for input polymer concentration c_0_ is proportional to the dimensionless area, 𝒜, between the tracer (the no-illite curve in Figure 2), and the polymer effluent, for a given case. The mass of adsorbed polymer is calculated as: m_ads_ = 𝒜.PV(in L).c_0_ (mg), which leads to an adsorption level at c_0_ of Γ = m_ads_/M_core_ (mg/g), where M_core_ is the mass of the core (in g). Thus, it appears that the adsorption in Figure 2 is “rate-dependent”, i.e., the adsorption level, Γ, has a different value at each flow rate. With this interpretation, Figure 2 indicates that polymer adsorption is higher at lower flow rates.

Other workers [20] have also observed this effect, even mentioning “rate-dependent polymer adsorption” in the title of their paper. In this paper, we note that at the higher flow rates, the fluid residence time in the core is only ~2 h.

Mousapour et al. [21] presented a recent series of polymer floods where the levels of adsorption were measured as described by measuring the difference, 𝒜, between polymer and tracer effluents, but again low fluid residence times were used.

There is an issue with all of the above dynamic core flood measurements of polymer adsorption, and in most similar studies in the literature. In particular, this effluent difference (𝒜) method is based on an important *assumption*; it is assumed that the adsorbing system is ***at equilibrium***. At equilibrium, when the effluent polymer concentration reaches the input level (c_0_), then the adsorbed level (at c_0_) is unequivocally known (to some experimental accuracy). However, if the system is not at equilibrium, the material balance simply tells us how much polymer (mass) is left in the core, but it does not necessarily mean that it is all adsorbed. At any value of effluent polymer concentration for this latter case, the in situ polymer may be in the process of adsorbing; and therefore, a lower value of adsorption level Γ will be estimated.

### 1.3. Aims and Objectives of This Paper

As noted above, kinetic effects have been suspected as being at least part of the explanation of polymer effluent behaviour in some pack and core floods [19]. In this work, it is shown how such effects might arise and how they might explain this polymer effluent tailing in polymer transport through porous rock cores or packed beds. The findings of this paper are then used to propose improved experimental procedures for both interpreting and quantifying polymer adsorption in the presence of kinetic adsorption effects. Also, procedures are proposed for proving whether the observed polymer effluent effects are actually due to kinetic phenomena or other possible causes.

The 3 main objectives of this paper are:

(i) To present a polymer transport and kinetic adsorption model which is appropriate for modelling polymer core and pack floods, including and accurately modelling the physics and kinetics of polymer adsorption described in this paper;
(ii) To apply this model to predict the qualitative behaviour of polymer core flood effluents in comparative equilibrium and non-equilibrium (kinetic) cases and to compare these results to experimental results published in the literature, and indeed to *explain* these observed results;
(iii) To use our results to propose improved experimental procedures for both interpreting and quantifying polymer adsorption in the presence of non-equilibrium (kinetic) effects. Clear procedural methods are presented for demonstrating that the observed polymer effluent effects are due to kinetic phenomena, as we claim.

## 2. The Freundlich Isotherm—Equilibrium and Kinetic Aspects

### 2.1. Origins of the Equilibrium Freundlich Model

**Figure 4:**
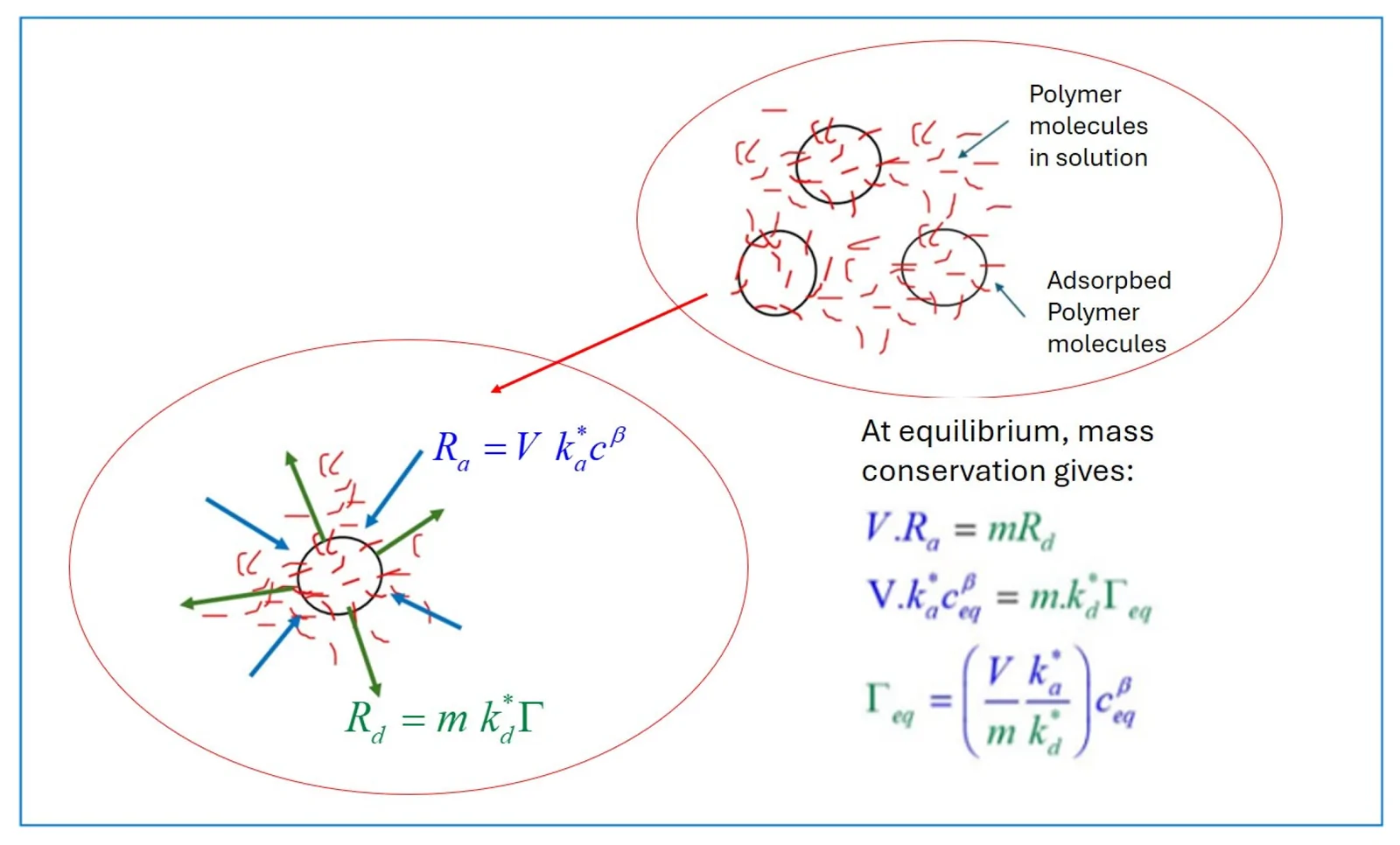
A schematic view of the kinetic processes of polymer adsorption onto the solid surface and desorption from this surface, showing the assumed expressions in the Freundlich model for *R_a_* and *R_d_*, the adsorption and desorption rate laws.

In this work, we will assume a Freundlich adsorption isotherm for the polymer/substrate equilibrium system. One feature of the Freundlich form that makes it appropriate for polymer adsorption is that it has a very steeply rising isotherm at concentrations just above c = 0. We note that the same work has been carried out for the Langmuir isotherm and the final conclusions and predictions are very similar to those from this paper, so the precise analytical equation of the isotherm is not of major importance. The equilibrium Freundlich isotherm, as a simple empirical form, is given by [1,4,6,7]:

Γ(*c*) = *α*.*c^β^* (3)

where α and β in Equation (3) are constant for a given set of conditions as listed above. The units used in this work are such that polymer concentration, c, is in mg/L, Γ is in mg/g, giving units for α as mg^(1−β)^L^β^g^−1^ and β is dimensionless. However, like many adsorption isotherms, the Freundlich model is based on an underlying view of the kinetics of the adsorption and desorption processes, and how this arises is outlined in Figure 4, where the schematic graphic relates specifically to the local process at pore/mineral grain scale. Like Figure 1, the schematic in Figure 4 relates exactly to the experimental process occurring at the fluid/mineral grain surface.

Figure 4 shows the assumed adsorption and desorption rate laws in the Freundlich model. The rate of adsorption from the bulk fluid is given as *R_a_*, and the rate of desorption from the solid is denoted *R_d_*. The dimensions of the associated rate constants are as follows: k_a_* has units of (mg/L)^(1−β)^.(time units)^−1^, such that *R_a_* will be (mg/L).(time units)^−1^, and the dimensions of k_d_^*^ are (time units)^−1^, such that *R_d_* will be (mg/g).(time units)^−1^. Thus, to convert to common units of mass/time in the material balance, the modified rate law assumptions are [22]:

$$\begin{array}{lll} \mathrm{Adsorption}\;\mathrm{rate}:\; & {R^{\prime }}_{a}=Vk_{a}^{*}C^{\beta } & \;\mathrm{where}\;\mathrm{this}\;\mathrm{will}\;\mathrm{be}\;\mathrm{in}\;\mathrm{units}\;\mathrm{of}\;\mathrm{mass}/\mathrm{time}\;(e.g.\;\mathrm{mg}/\mathrm{hour})\\ \mathrm{Desorption}\;\mathrm{rate}:\; & {R^{\prime }}_{d}=mk_{d}^{*}\Gamma & \;\mathrm{also}\;\mathrm{in}\;\mathrm{the}\;\mathrm{same}\;\mathrm{units}\;\mathrm{of},\;\mathrm{mass}/\mathrm{time} \end{array}$$

As shown in Figure 4, at equilibrium then ${R^{\prime }}_{a}={R^{\prime }}_{d}$ and therefore, it is clear that in general:

(4) $$\Gamma (c)=\left(\frac{V.k_{a}^{*}}{m.k_{d}^{*}}\right)c^{\beta }$$

And hence the constant, α, in the Freundlich model is given by

(5) $$\alpha =\left(\frac{V.k_{a}^{*}}{m.k_{d}^{*}}\right)$$

**Figure 5:**
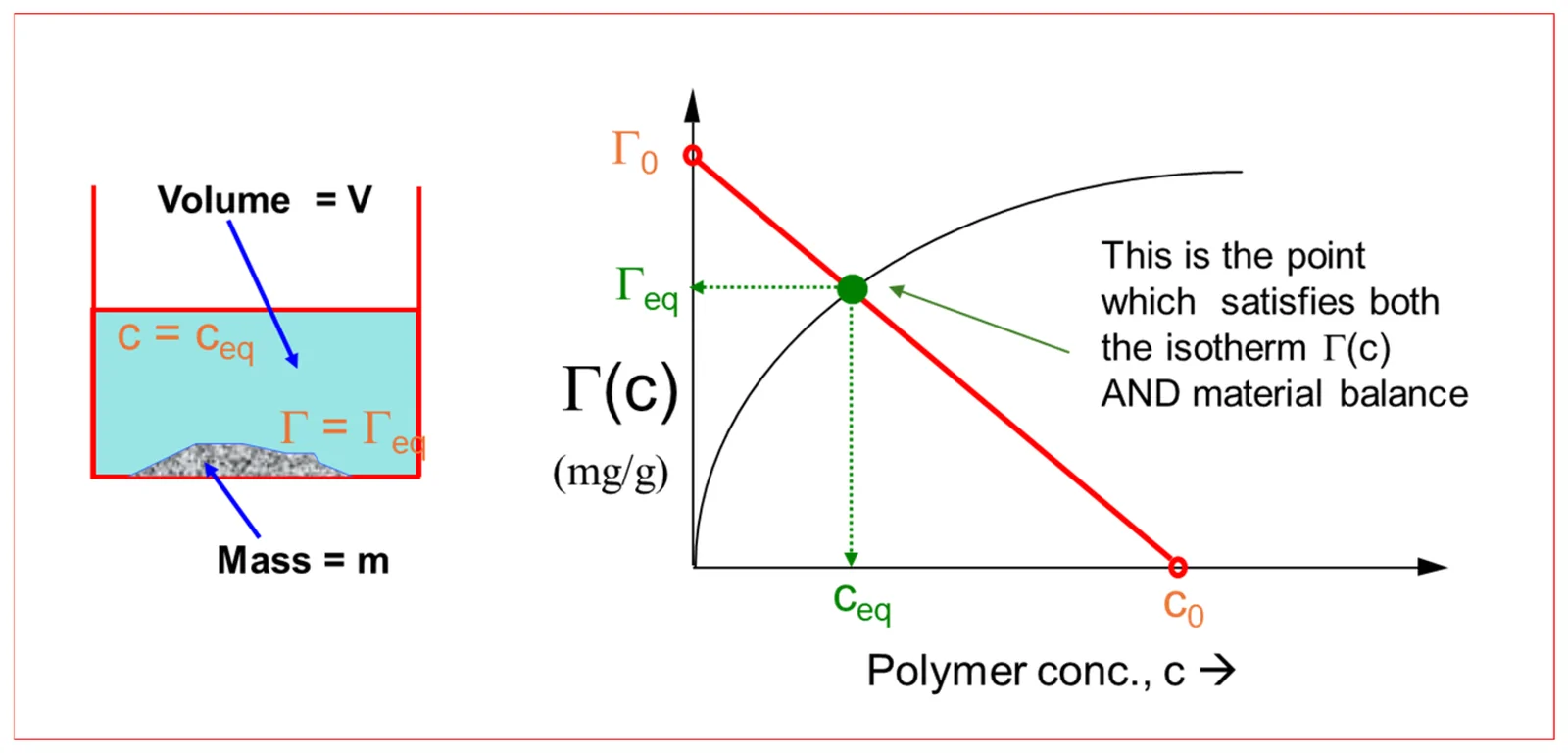
The construction which is applied (numerically) to calculate the equilibrium values of c_eq_ and Γ_eq_ in a system with given values of m, V and c_0_ and any assumed analytical form of the adsorption isotherm, Γ(c).

It may appear unusual here to have the factor (V/m) as part of the Freundlich constant α, but this is of particular use in the work presented here, and Equation (3) still applies with this value of α for *any* V/m ratio, as we will show below. In the derivation of the Freundlich isotherm in Figure 2, we could have directly equated the quantities *R_a_* and *R_d_*; *R_a_* = *R_d_* implies that $k_{a}^{*}c^{\beta }=k_{d}^{*}\Gamma$ which would lead to the “primitive” form of the Freundlich adsorption isotherm as $\Gamma^{\prime }=\left(k_{a}^{*}/k_{d}^{*}\right)c^{\beta }=\alpha^{\prime }c^{\beta }$, where the *α*^′^constant is given by $\alpha^{\prime }=\left(k_{a}^{*}/k_{d}^{*}\right)$; a closer examination of the quantity *Γ*^′^ shows that it has units of concentration (shown below). This makes no reference to the (V/m) ratio, but this form is not a proper adsorption isotherm since the units must incorporate implied m and V since it does not give the adsorption in mg/g, as required. The relation between the form used in this work and this “primitive” form can be seen when we use this equation to calculate both the equilibrium adsorption and concentration levels, i.e., Γ_eq_ and c_eq_, as shown in Figure 1, where we know the actual isotherm, i.e., Γ = *αc^β^*. In such a calculation, we know m, V and the initial polymer concentration, c_0_, and we must find both Γ_eq_ and c_eq_. The construction to perform this calculation is shown schematically in Figure 5, which relates the material balance in the system directly to the experimental adsorption experiment. Clearly, the mass of polymer initially in the system is Vc_o_ (in mg), and at equilibrium this mass is distributed between the adsorbed polymer (mΓ_eq_) and the polymer in solution (Vc_eq_), and mass conservation requires that Equation (6) applies:

Mass of polymer in system = *Vc*_0_ = mΓ*_eq_*(*c_eq_*) + *V*.*c_eq_* (6)

However, since we know the equation for the Freundlich isotherm and the values of the α and β parameters, to find c_eq_ we must solve the equation:

F(c*_eq_*) = mΓ*_eq_*(*c_eq_*) + *V*.*c_eq_* − *Vc*_0_ = 0 (7)

Dividing Equation (7) by V and using Equation (A5) gives that:

(8) $${F(c}_{\mathrm{eq}})=\alpha^{\prime }c_{\mathrm{eq}}^{\beta }+c_{\mathrm{eq}}-c_{0}=0$$

where the first term in Equation (8) is the primitive form Γ^′^ = *α*^′^*c^β^*, and this can be used to determine the equilibrium concentration (c_eq_) arising from any initial c_0_ value by numerically solving Equation (8) for c_eq_. The corresponding adsorption level, Γ (in mg/g), is then found by calculating $\Gamma =\left(m/V\right)\alpha^{\prime }c_{\mathrm{eq}}^{\beta }=\alpha c_{\mathrm{eq}}^{\beta }$.

### 2.2. The Full Kinetic Adsorption Model

The full kinetic model is now considered to describe the kinetics of the change in the (increasing) adsorption level, Γ, and that of the (decreasing) solution concentration level. Clearly, Γ increases as c decreases until equilibrium is reached at Γ = Γ_eq_ and c = c_eq_. These 2 equations are of course closely related and written as follows [22]:

(9) $$\begin{array}{llll} & \mathrm{adsorption} & \text{-}\mathrm{desorption} & \\ \left(\frac{d\Gamma }{\mathrm{dt}}\right)= & +\frac{k_{a}^{*}Vc^{\beta }}{m}\;- & \frac{k_{d}^{*}m\Gamma }{m} & \;;\;\Rightarrow \left(\frac{d\Gamma }{\mathrm{dt}}\right)=+\left(\frac{k_{a}^{*}Vc^{\beta }}{m}\;\right)-k_{d}^{*}\Gamma \end{array}$$

(10) $$\left(\frac{\mathrm{dc}}{\mathrm{dt}}\right)=-\frac{k_{a}^{*}Vc^{\beta }}{V}+\frac{k_{d}^{*}m\Gamma }{V}\;;\;\Rightarrow \left(\frac{\mathrm{dc}}{\mathrm{dt}}\right)=-k_{a}^{*}c^{\beta }+\left(\frac{k_{d}^{*}m\Gamma }{V}\right)$$

**Figure 6:**
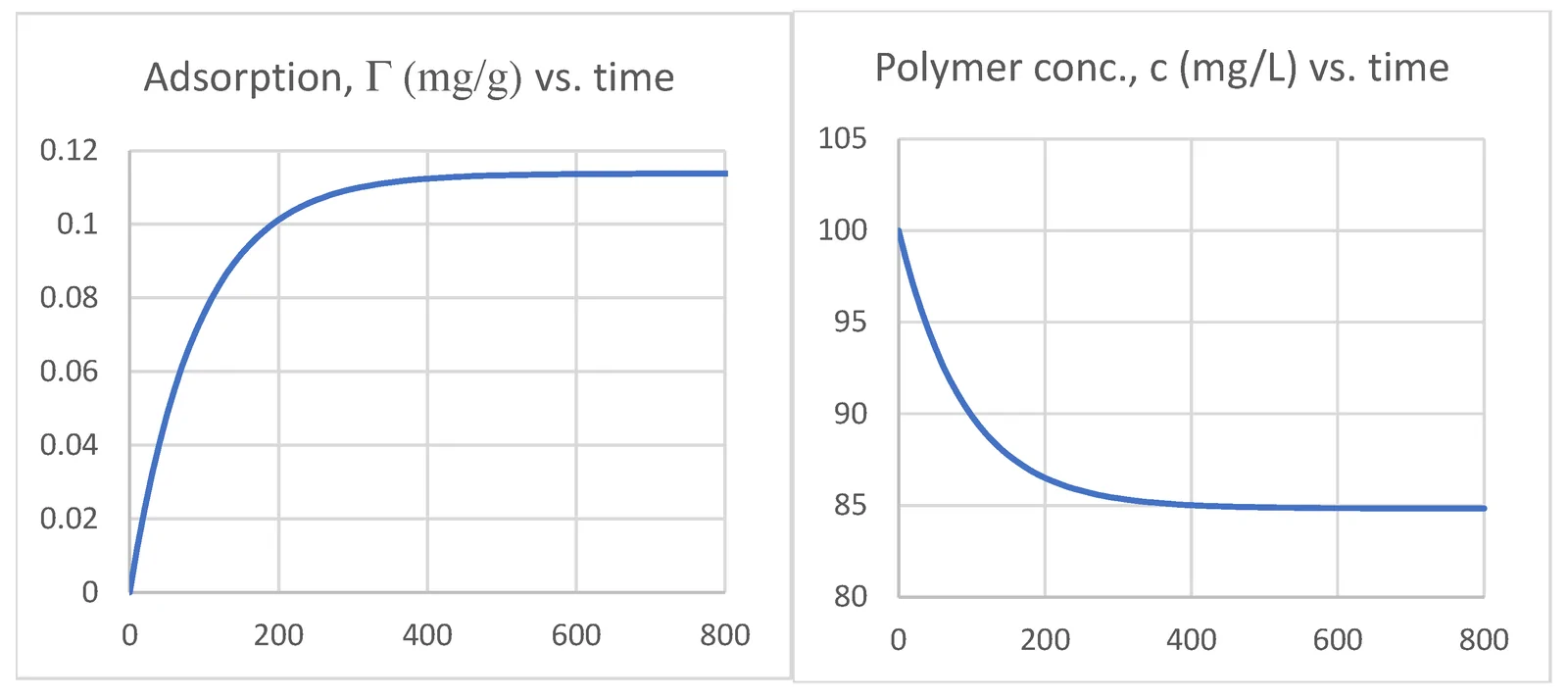
The calculated *Γ* vs. *t* and *C* vs. *t* for the numerical example in Table 1. The time here is in hours but can be minutes or seconds according to the time scale required, where the units in Table 1 are changed accordingly.

These coupled ordinary differential equations (ODEs) are not soluble analytically, except under simplified conditions as shown below. However, to illustrate how they operate, a simple numerical example is presented using the model data in Table 1. Using this data, Equations (9) and (10) can be integrated numerically to obtain the profiles of Γ vs. t and C vs. t, as shown in Figure 6. From the numerical integration of the full kinetic equations above, the final equilibrium level of adsorption was found to be Γ_eq_ = 0.11380 mg/g and the concentration was C_eq_ = 84.82593 mg/L (Note that the numbers quoted here (and elsewhere in this paper) are very “over accurate” in that they could not be determined to this accuracy experimentally. They are quoted in this way for computational reasons, i.e. for checking the numerical calculations). This can be checked by substituting the value of c_eq_ into the Freundlich model with α = 0.0375, β= 0.25 (in the units of Table 1); this gives exactly Γ_eq_ = 0.11380 mg/g, as required.

### 2.3. The Simplified Kinetic Freundlich Model

As a simplification, consider only the *adsorption* term in the kinetics of polymer adsorption, as follows (Equation (9)) [22]:

(11) $$\begin{array}{lll} & \mathrm{adsorption} & \mathrm{desorption}\\ \left(\frac{d\Gamma }{\mathrm{dt}}\right)= & +\left(\frac{k_{a}^{*}Vc^{\beta }}{m}\;\right)- & k_{d}^{*}\Gamma \end{array}$$

Clearly, the problem with solving this kinetic equation is that there is a “c” in the adsorption term on the right-hand side of the equation. This concentration term is changing (reducing) with t, as shown in the results for the full kinetic model in Figure 6 above. But suppose we make the further assumption that concentration, c, is set to *c* = *c*_0_, i.e., the maximum or initial input concentration. This would mean that the term $\left(\frac{k_{a}^{*}Vc_{0}^{\beta }}{m}\;\right)$ is no longer changing but is a constant. From Equation (5), this term is clearly:

(12) $$\left(\frac{k_{a}^{*}Vc_{0}^{\beta }}{m}\;\right)=k_{d}^{*}\alpha c_{0}^{\beta }=k_{d}^{*}\Gamma_{\max}$$

where Γ_max_ is the highest level of adsorption that can be reached if c = c_0_, and this allows us to write Equation (11), as follows:

(13) $$\left(\frac{d\Gamma }{\mathrm{dt}}\right)=\;k_{d}^{*}\Gamma_{\max}-k_{d}^{*}\Gamma =k_{d}^{*}\left(\Gamma_{\max}-\;\Gamma \right)$$

This simple ODE is actually a standard form which has a well-known solution, which is easily shown to be:

(14) $$\Gamma (t)=\Gamma_{\max}\left(1-e^{-k_{d}^{*}t}\right)$$

**Figure 7:**
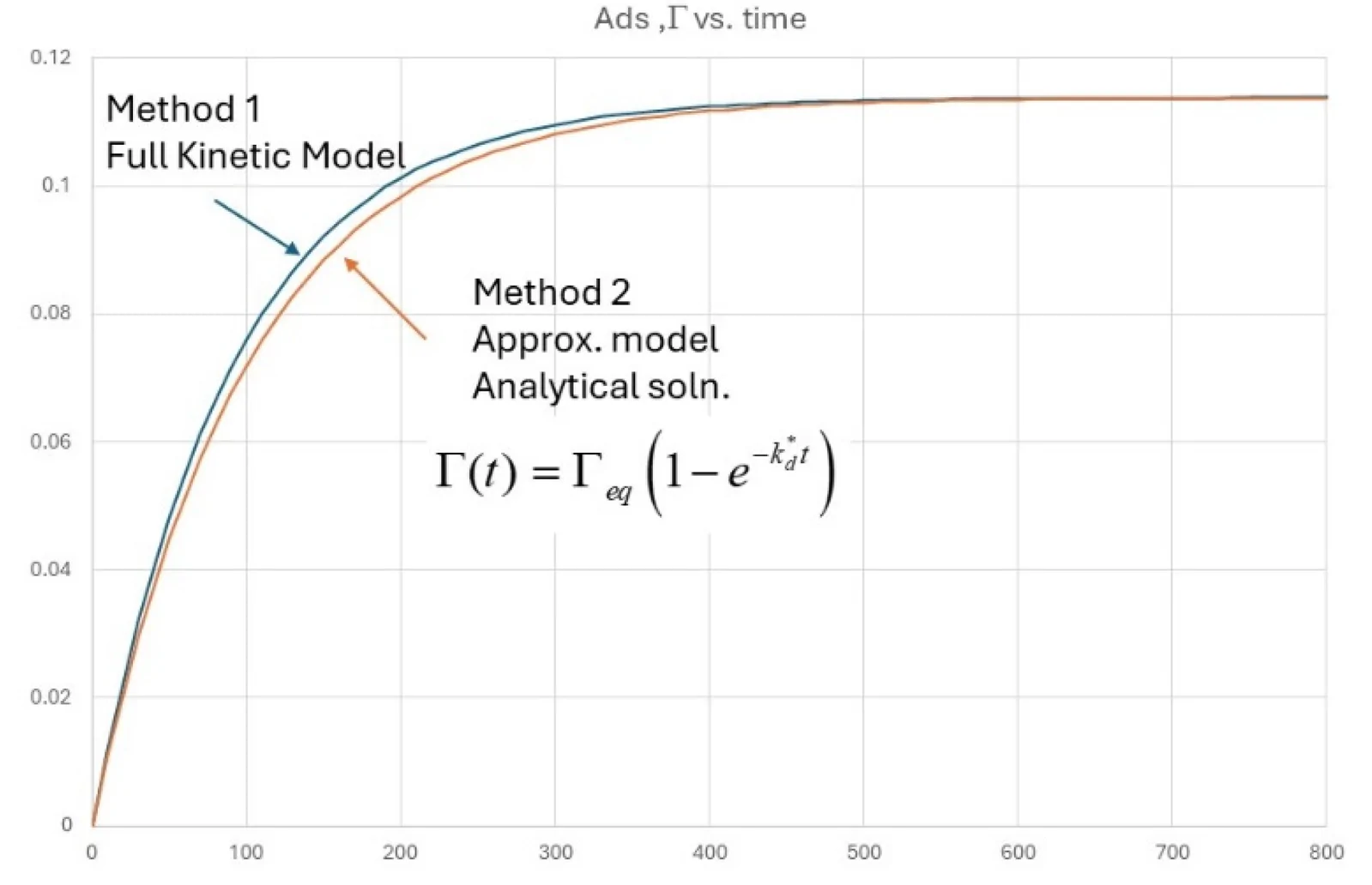
Comparison of the polymer adsorption, Γ vs. time (h) for the data in Table 1 using the full integration of Equations (9) and (10) and the approximate solution of Equation (14).

The full system of equations shown in Section 2.2 above had a final polymer concentration of c_eq_ = 84.83 mg/L, giving a final adsorption level of Γ_eq_ = 0.114 mg/g. In the approximate model where we set c = 100 ppm (i.e., the initial fixed concentration, c_0_), then Γ_max_ = 0.119 mg/g (using the Freundlich parameters in Table 1). These final adsorption levels are quite close, and we may therefore ask: how do the results of the approximation in Equation (14) compare with the full kinetics given in Section 2.2 above (i.e., the numerical integration of Equations (9) and (10))? The two methods (1. the full kinetic and 2. the approx. model) are compared in Figure 7 for the data given in Table 1 above. The agreement, as shown in Figure 7 (with kd* = 0.01 h^−1^), is seen to be very good, and probably much less than the experimental error in the polymer assay.

## 3. The Polymer Transport and Kinetic Adsorption Equations

Polymer transport through porous media in 1D, including kinetic adsorption using the Freundlich-based model, is described by the modified convection-dispersion equations as follows [1,23]:

(15) $$\left(\frac{\partial c}{\partial t}\right)=D\left(\frac{\partial^{2}c}{\partial x^{2}}\right)-v\left(\frac{\partial c}{\partial x}\right)-\frac{\rho }{\phi }\left(\frac{\partial \Gamma }{\partial t}\right)$$

(16) $$\left(\frac{\partial \Gamma }{\partial t}\right)=\left(\frac{Vk_{a}}{m}\right)c^{\beta }-k_{d}\Gamma$$

where D is the dispersion coefficient, ν is the pore velocity of the fluid (*v* = *q*/(*ϕA*)), q is the volumetric flow rate, ϕ is the porosity, and A is the cross-sectional area of the 1D system of length L (a core or sand pack, for example); ρ is the rock density and the other quantities associated with polymer adsorption are described previously. Equations (15) and (16) are in the laboratory units introduced earlier in this paper.

The complete corresponding set (to Equations (15) and (16)) of *dimensionless* equations for polymer transport and kinetic adsorption, derived in Appendix A, is as follows:

(17) $$\left(\frac{\partial C}{\partial T}\right)=\frac{1}{N_{\mathrm{Pe}}}\left(\frac{\partial^{2}C}{\partial X^{2}}\right)-\left(\frac{\partial C}{\partial X}\right)-N_{\mathrm{Ad}}\left(\frac{\partial \tilde{\Gamma }}{\partial T}\right)$$

and

(18) $$\left(\frac{\partial \tilde{\Gamma }}{\partial T}\right)=N_{\mathrm{Da}\_A}\left[C^{\beta }-\tilde{\Gamma }\right]$$

where $C=\frac{c}{c_{o}};X=\frac{x}{L};T=\frac{v.t}{L};\tilde{\Gamma }=\frac{\Gamma }{\Gamma_{\max}}$ are the dimensionless concentration, length, time and adsorption level, respectively, and $0\leq C\leq 1\;\mathrm{and}\;0\leq \tilde{\Gamma }\leq 1$. The 3 dimensionless groups governing the system are the Peclet number, *N_Pe_*, the Adsorption number, *N_Ad_*, and the adsorption Damköhler number, *N_Da_*__*A*_, which are given by the expressions in Equation (19) below (see Appendix A):

(19) $$N_{\mathrm{Pe}}=\frac{\mathrm{vL}}{D};\;N_{\mathrm{Ad}}=\left(\frac{\rho \Gamma_{\max}}{{\phi c}_{o}}\right)\;\mathrm{and}\;N_{\mathrm{Da}\_A}=\left(\frac{Lk_{d}^{*}}{v}\right)$$

Since the transport is convection-dominated, the Peclet number is generally relatively high (*N_Pe_* > 80) and is not of great importance in this system; we have checked this by simulation, but no further details are given here. However, the other two numbers, the Adsorption number, *N_Ad_* and the adsorption Damköhler number, *N_Da_*__*A*_, are both very important, as will be demonstrated below. *N_Ad_* quantifies the degree of retardation of the polymer front for a system at *equilibrium* adsorption, and is often called the retardation factor, R. *N_Da_*__*A*_ expresses the balance between the residence time of the polymer in the system (i.e., *L*/*v*) to the timescale of kinetic adsorption (i.e., $1/k_{d}^{*}$). As expected, long residence times (low velocities) and fast adsorption kinetics (i.e., high values of *N_Da_*__*A*_) favour equilibrium, or near-equilibrium, conditions; whereas low residence times (high velocities) and slow adsorption times (i.e., low values of *N_Da_*__*A*_) lead to highly non-equilibrium kinetic-dominated behaviour.

We again stress that although the model and results presented here are based on the underlying equilibrium and kinetic assumptions of the Freundlich adsorption model, the qualitative findings are true for any “normal” (concave down) analytical adsorption isotherm. Indeed, we take as a common assumption that the equilibrium polymer adsorption isotherm is steeply rising at low concentrations, is concave downwards and has a maximum adsorption level or a plateau adsorption level (Γ_max_). We also note (but do not strictly assume) that this plateau adsorption level may occur at a relatively “low” polymer concentration level of c ≈ 50–500 mg/L. Numerical simulation examples will be presented to illustrate the extent to which the variation in where the plateau occurs impacts the main results and findings of this work. The transport and kinetic equations (Equations (6) and (15), or Equations (17) and (18)) are solved numerically using standard finite difference methods. 

## 4. Numerical Results for Polymer Transport for Equilibrium and Kinetic Polymer Adsorption

In order to achieve the objectives outlined in Section 1 above, we present a series of simulations addressing various aspects of the polymer transport/kinetic polymer adsorption process.

### 4.1. Context of the Numerical Examples and Exact N_Da_A_ Scaling

The entire range of possible results which can arise for any mathematical model of a given polymer transport and kinetic adsorption model, in a 1D core system (e.g., a sandstone core or mineral pack) ranges from equilibrium adsorption (as *N_Da_*__*A*_ → *∞*), to effectively no adsorption (as *N_Da_*__*A*_ → 0), the latter case being described as “tracer like” behaviour. When the system is at equilibrium, the polymer adsorption rate is very high relative to the polymer fluid residence time. Otherwise, the adsorption rate is either comparable to or longer than the residence time. To systematically vary the adsorption Damköhler number, there are two exactly scaled equivalent choices (this exact scaling demonstrated below and in Appendix A):

(i) we can choose a fixed flow velocity (ν) and then systematically vary the adsorption rate constant $k_{d}^{*}$ (remembering to change $k_{a}^{*}$ in exact proportion to keep α constant), or
(ii) we may take a constant kinetic rate, $k_{d}^{*}$, and then vary the flow rate (ν) from a very low to a high value.

Numerical examples are presented below which demonstrate that these equivalent cases scale exactly.

The work in this paper was stimulated by two relatively recent sets of experimental observations:

(a) To investigate whether the effluent “tailing” behaviour measured in the experiments of Seright and coworkers was due to the kinetic adsorption behaviour of the polymer [17,18,19] or possibly some other effect; and
(b) To study the consequences of the recent observations by Silva and coworkers that HPAM polymer adsorption at lower temperatures (T ~30 °C) could take relatively long times to reach equilibrium for sand in beakers; i.e., several days [15,16]. Again, we caution that beaker tests may give exaggerated (i.e., too long) experimental adsorption time scales (i.e., too low values of k_d_*), for the reasons discussed above.

**Figure 8:**
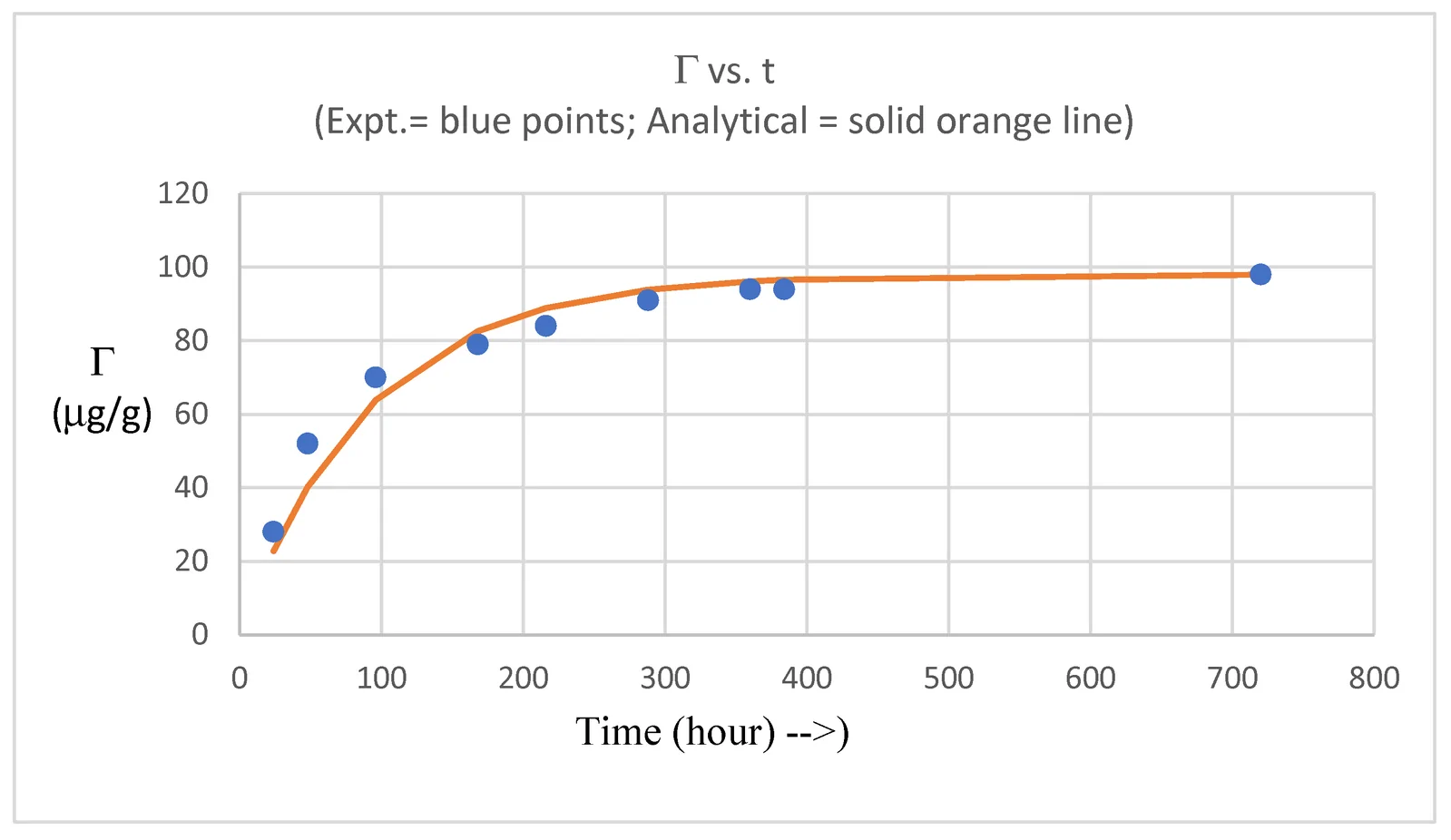
The adsorption of HPAM polymer (Γ) vs. time onto quartz sand. Experimental data (blue points), matched using the model of Equation (10) (solid line). Data of Silva et al. [14,15].

To construct numerical examples that are relevant to recent experimental results, we took one of the typical observations of Silva et al. [15,16] based on static bulk adsorption tests, as shown in Figure 8. This figure presents a plot of HPAM adsorption measurements (shown as ●) over time (in hours) onto quartz sand at T = 31 °C, and these data are matched by fitting Equation (14), in which Γ_max_ = 98 μg/g and $k_{d}^{*}$ = 0.01 h^−1^. The match is not perfect, but it gives the correct order of magnitude of the $k_{d}^{*}$ and this is all we require for our simulations. To contextualise this value of the adsorption rate ($k_{d}^{*}$ = 0.01 h^−1^), recall that the adsorption “half-life” (i.e., the time to get to dimensionless adsorption $\tilde{\Gamma }$ = (Γ/Γ_max_) = 0.5) is given by $\tau_{1/2}=-\ln\left(0.5\right)/k_{d}^{*}$ ≈ 69 h (~2.9 days), and to reach $\tilde{\Gamma }$ = 0.95, the $\tau_{0.95}$ ≈ 300 h (~12.5 days).

The polymer adsorption literature does note that the whole adsorption process, including stages (i)–(v) briefly reviewed in Section 1, may take “hours to days” [4]. However, it is reasonable to ask for the specific results here: how much of the long time (~300 h) taken for polymer adsorption shown in Figure 8 is taken up by diffusion of the polymer through the bulk and then into the porous medium (an unconsolidated sand)? In other words, is the rate constant (k_d_*) which we derive here too low at the value k_d_* = 0.01 h^−1^? In fact, this precise number may indeed be too low to directly apply in a porous medium, but to be sure of this we would need to compare this result with experiments where full system stirring or agitation took place to ensure constant contact of the surface with the polymer solution (experiments currently in progress). However, using this number (k_d_* = 0.01 h^−1^) does *not* affect our conclusions, since we demonstrate below that the results depend only on the adsorption Damköhler number (N_Da-A_). This quantity is the ratio of the residence time of the polymer in the core, (L/ν), to the timescale of the adsorption process, (1/k_d_*). Thus, simulated polymer effluent results for kinetic polymer adsorption at *any* value of k_d1_* and fluid velocity ν_1_ are the same at any other value, such as k_d2_*, but for an adjusted flow velocity in the core, ν_2_ = ν_1_ (k_d2_*/k_d1_*), since they have the same value of N_Da-A_. This exact scaling is demonstrated below.

### 4.2. The Numerical Examples

**Figure 9:**
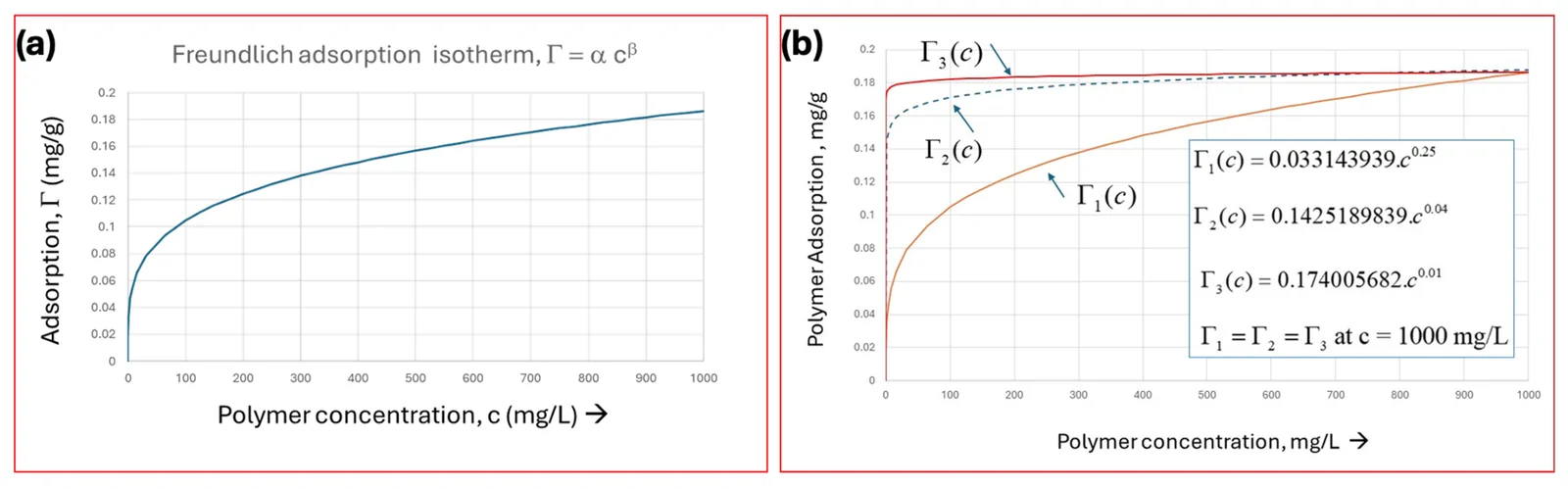
(**a**) The base Freundlich adsorption isotherm (Γ_1_) for the kinetic parameters in Table 2; α = 0.03314 and β = 0.25, such that for c in mg/L, adsorption Γ is in mg/g; (**b**) two more sharply rising “stripping” Freundlich adsorption isotherms (Γ_2_ and Γ_3_) compared with the base case (Γ_1_) are used in later sensitivities. The “over accurate” numerical coefficients describing these analytical forms are provided to allow exact numerical comparison to be carried out by other researchers.

In this work, a 1D core flood is simulated with the physical properties listed in the first two columns of Table 2. The numerical grid used for the simulations is given in the next two columns, and the base case specifications of the polymer properties are given in the final two columns. Note that the manner in which the polymer input data is presented implies that we input the kinetic adsorption parameters $k_{a}^{*}\;\mathrm{and}\;k_{d}^{*}$ and then calculate the value of the Freundlich constant, α. In practice, however, we actually first decide on the equilibrium Freundlich isotherm parameters (α and β in Equation (2)), with units such that if polymer concentration c is in mg/L, then the adsorption level is in mg/g of rock. The quantity $k_{d}^{*}$ is chosen based on kinetic adsorption experiments as shown in Figure 8, and then the corresponding $k_{a}^{*}$ quantity is calculated using Equation (5) as $k_{a}^{*}=\left(\frac{\mathrm{\alpha m}.k_{d}^{*}}{V}\right)$. For the parameters in Table 2, the base case Freundlich isotherm, Γ_1_, is shown in Figure 9a, and two other more steeply rising isotherms, Γ_2_ and Γ_3_, used in later sensitivities, are also shown in Figure 9b.

As noted above, a complete set of cases spanning the entire range of physical possibilities from equilibrium adsorption (N_Da-A_ → ∞) to almost “tracer-like” behaviour (N_Da-A_ → 0) can be constructed by choosing a single rate parameter (k_d_*) and then varying the fluid volumetric flow rate (q) (i.e., the velocity, ν = q/(Aϕ)). The range of examples for which results are presented below is listed in Table 3, where the types of behaviour listed from “near equilibrium” to “almost tracer-like” are anticipated and will be explained as the results are discussed below.

### 4.3. Continuous Injection Polymer Equilibrium and Kinetic Adsorption Cases

The first set of numerical results is shown for continuous injection of several PV (6–12) of polymer solution at a concentration of c_0_ = 1000 mg/L into the core. Continuous injection simulations for all of the cases in Table 3 have been carried out.

**Figure 10:**
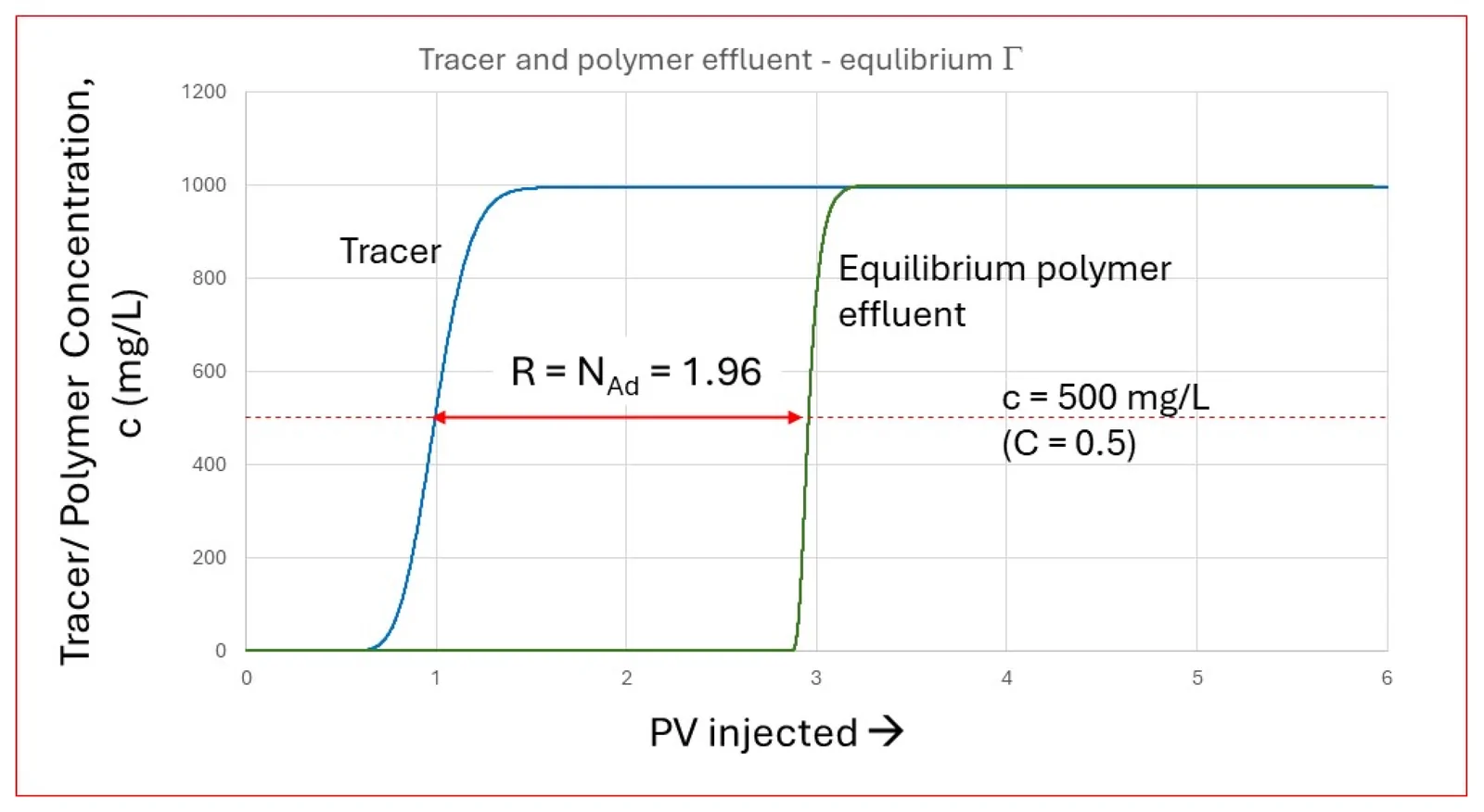
The simulated effluent curves of a non-adsorbing tracer and the polymer at equilibrium adsorption. These 2 cases are virtually identical to Case 1 in Table 3 (equilibrium adsorption Γ_1_, according to the isotherm in Figure 9) and Case 16 (which has such a low adsorption Damköhler number that the polymer scarcely adsorbs, apparent Γ ≈ 0, over the time of this displacement flood).

*“End Member” Cases—Equilibrium and Tracer:* Before presenting the range of non-equilibrium cases, we first consider the “end member” cases of equilibrium adsorption (N_Da-A_ → ∞) and almost no adsorption (N_Da-A_ → 0; tracer-like behaviour). The effluent curves for these two cases are shown in Figure 10. An analytical prediction explained in Appendix A, is that the retardation (R = N_Ad-A_) of the equilibrium polymer is R = 1.96 (PV), and this is what is calculated numerically (see Figure 10). A second point to note is that the equilibrium polymer breakthrough effluent is somewhat steeper or sharper than the tracer effluent curve. This behaviour is well known as the concave-downwards adsorption isotherm is self-sharpening (p. 230, [1]). Since we have just referred to these two simulations as “end member” cases, we may expect that all the other “intermediate” effluent behaviours might show effluents lying between these two cases, but this is certainly not the case as is now shown.

**Figure 11:**
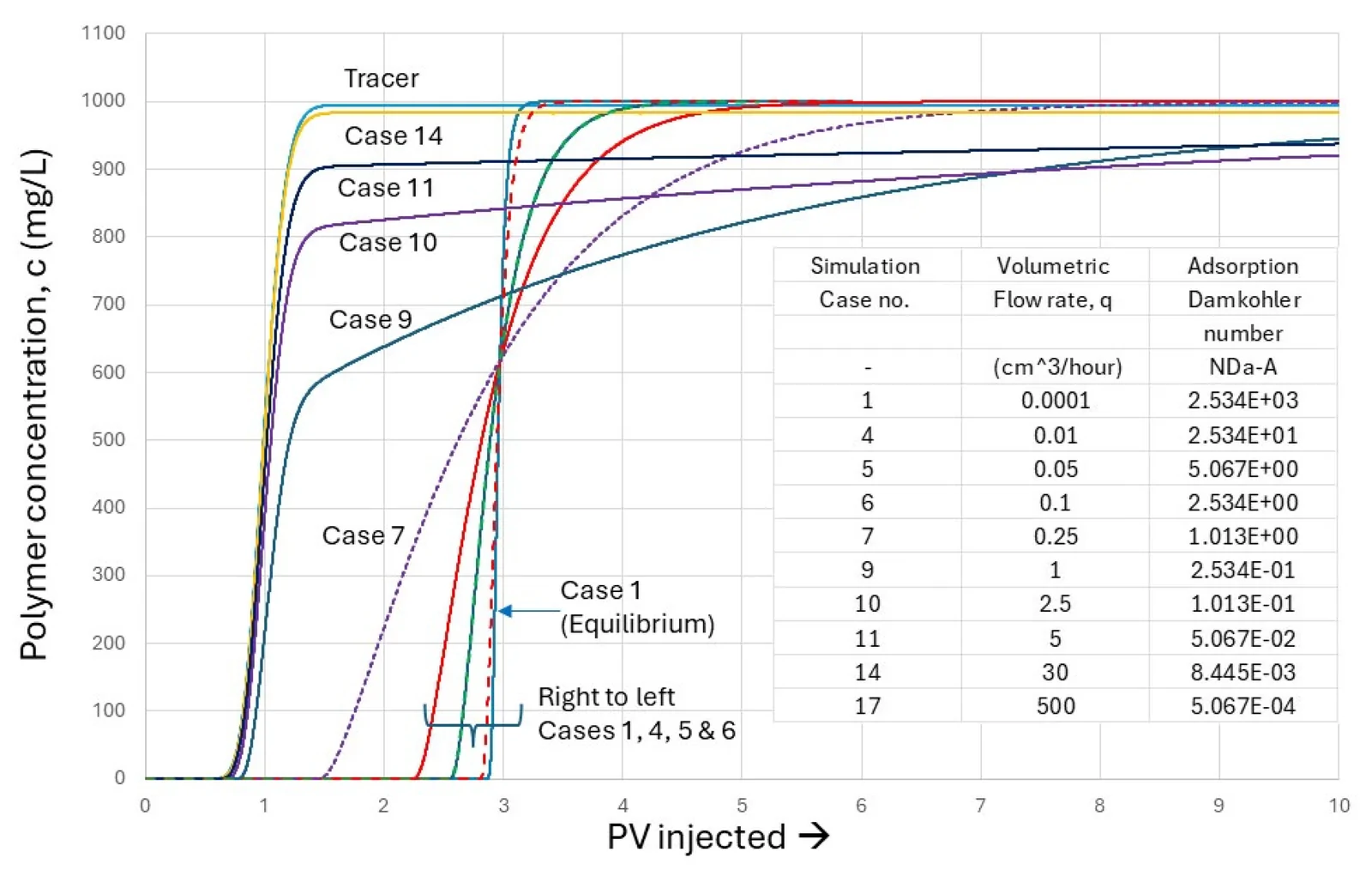
The simulated polymer effluents for a wide range of selected cases from Table 3 showing the span of predicted behaviours from very high adsorption Damköhler numbers, N_Da-A_ (effectively equilibrium; Case 1) to very low N_Da-A_ values (effectively tracer, Case 17).

*The Entire Range of Polymer Effluent Behaviours:* Figure 11 presents a selection of calculations covering the whole range of possible kinetic adsorption behaviours, between equilibrium adsorption and tracer-like cases. This figure shows a very wide range of behaviours, including many cases where the long “tailing” of the effluent goes well outside the domain bounded by the equilibrium and tracer cases in Figure 10. To unravel this complexity, we will now consider subsets of these cases under the headings:

- Near-equilibrium cases at *high* values of N_Da-A_;
- Transitional kinetics cases;
- Highly non-equilibrium cases (show the long-tailing behaviour quite clearly);
- Almost tracer-like cases (showing a plateau behaviour), N_Da-A_ → 0.

**Figure 12:**
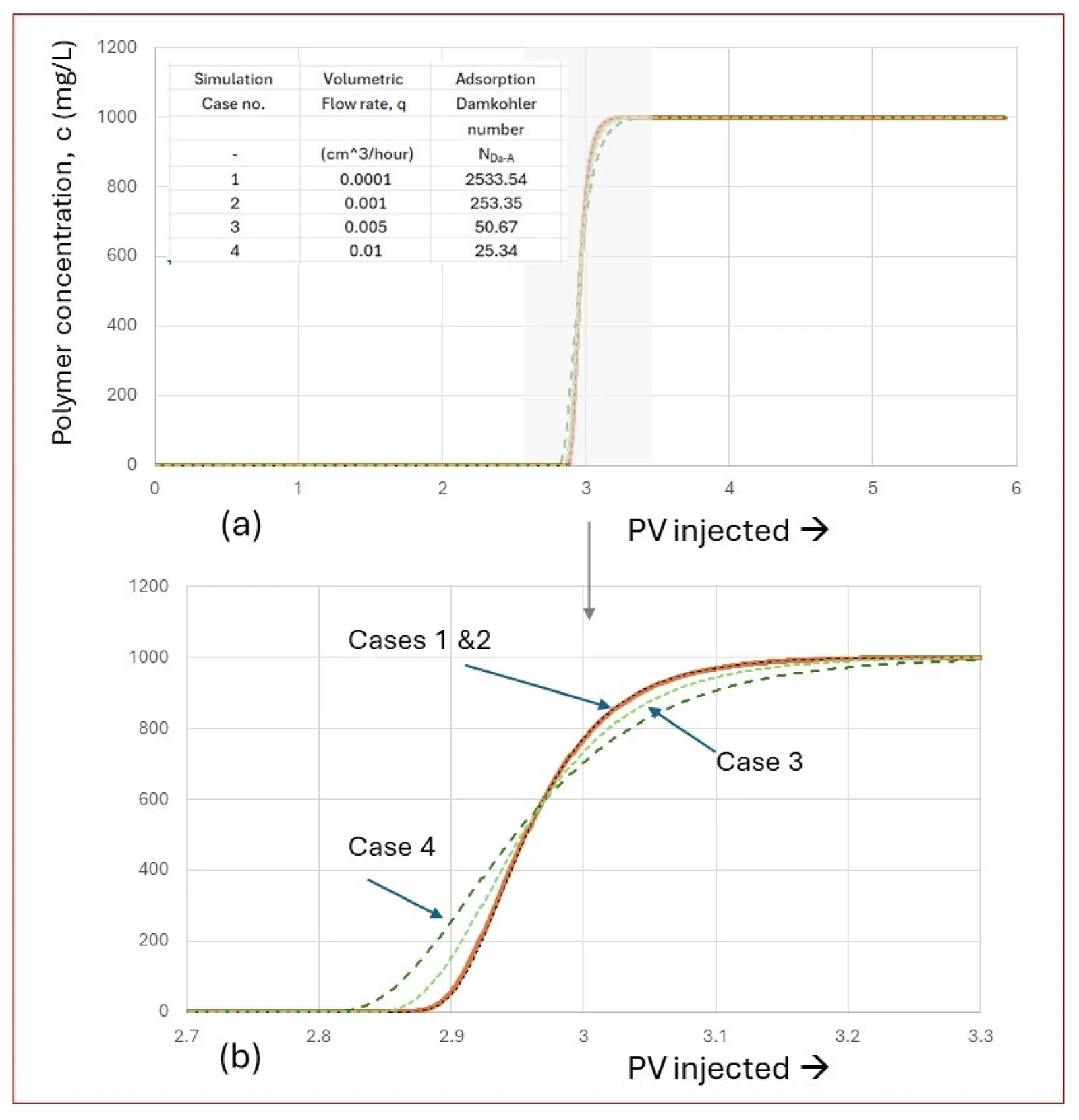
The simulated polymer effluent curve for the “near-equilibrium” Cases 1–4. (**a**) shows the effluents on the full PV scale and (**b**) shows the four effluents within the grey shaded area of (**a**) between 2.7 and 3.3 PV. In an experimental system, these cases would be almost indistinguishable when looking only at the continuous polymer injection effluents.

*Near Equilibrium Polymer Effluents (Cases 1–4;* Table 3*):* The near-equilibrium cases are shown in Figure 12a plotted over the entire flood (0–6 PV), and in Figure 12b plotted over the narrow range of the flood front (2.7–3.3 PV). Although a clear trend is visible over these four cases as the system moves gradually away from the full equilibrium cases, in practice, from an experimentally measured polymer concentration effluent viewpoint, these cases could probably not be distinguished analytically.

**Figure 13:**
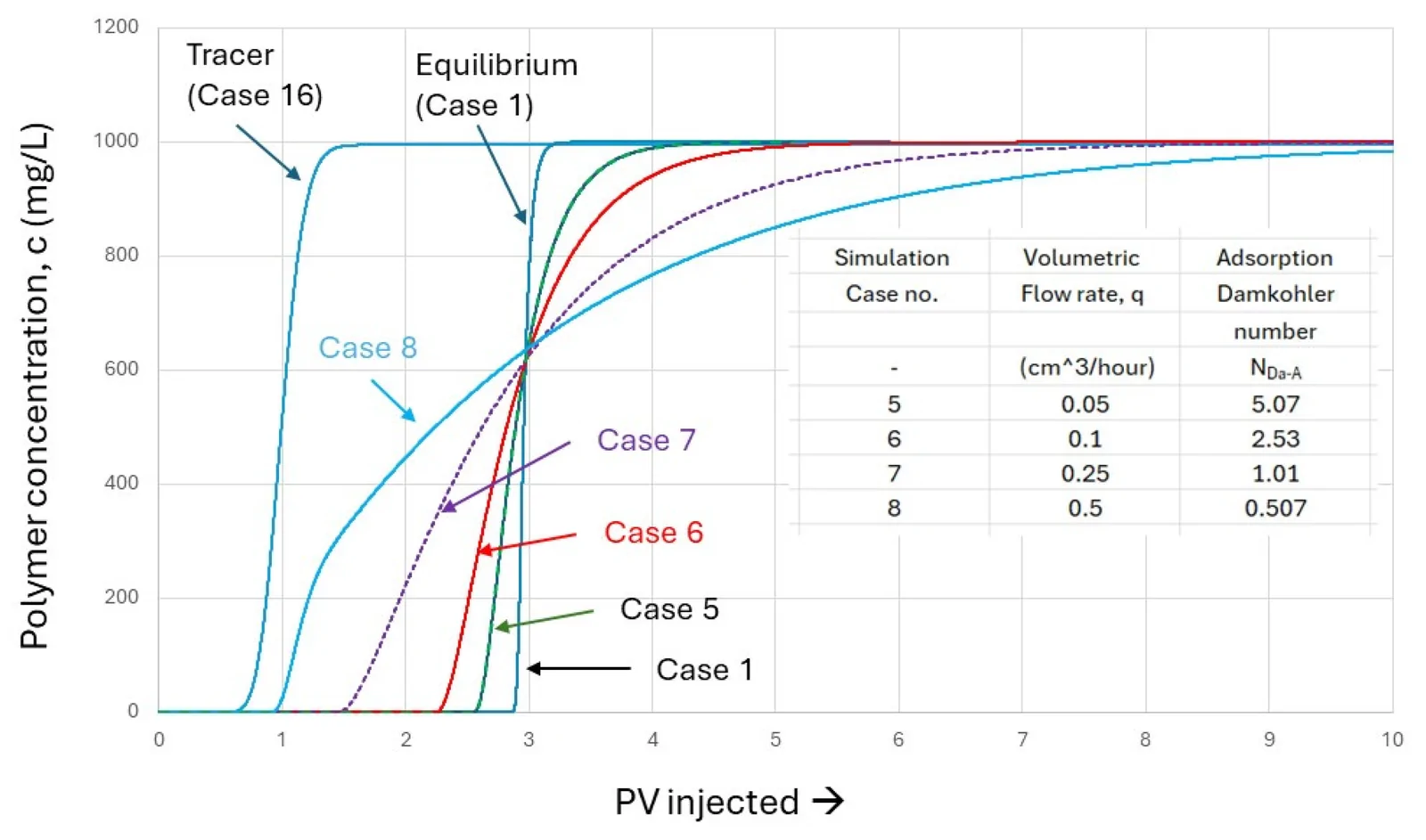
The simulated polymer effluent curve for the “transitional kinetic” Cases 5–8, where the differences from the equilibrium case (Case 1) are now very evident. These effluents would be clearly distinguishable experimentally from the expected equilibrium behaviour.

*The Transitional Kinetic Polymer Effluents (Cases 5–8;* Table 3*):* The transitional kinetics cases are shown in Figure 13 plotted over the entire flood (0–10 PV). These cases would be quite distinct from the equilibrium effluent, especially Case 8, which shows very significant “tailing” where the effluent does not reach the input polymer concentration (c_0_) until over 10 PV of injection. Cases 6–8 show that the non-equilibrium effect causes the breakthrough time to become rather earlier, moving quite close to the tracer breakthrough time (see Figure 10). This feature will be even more marked as the system moves further from equilibrium in the following cases.

**Figure 14:**
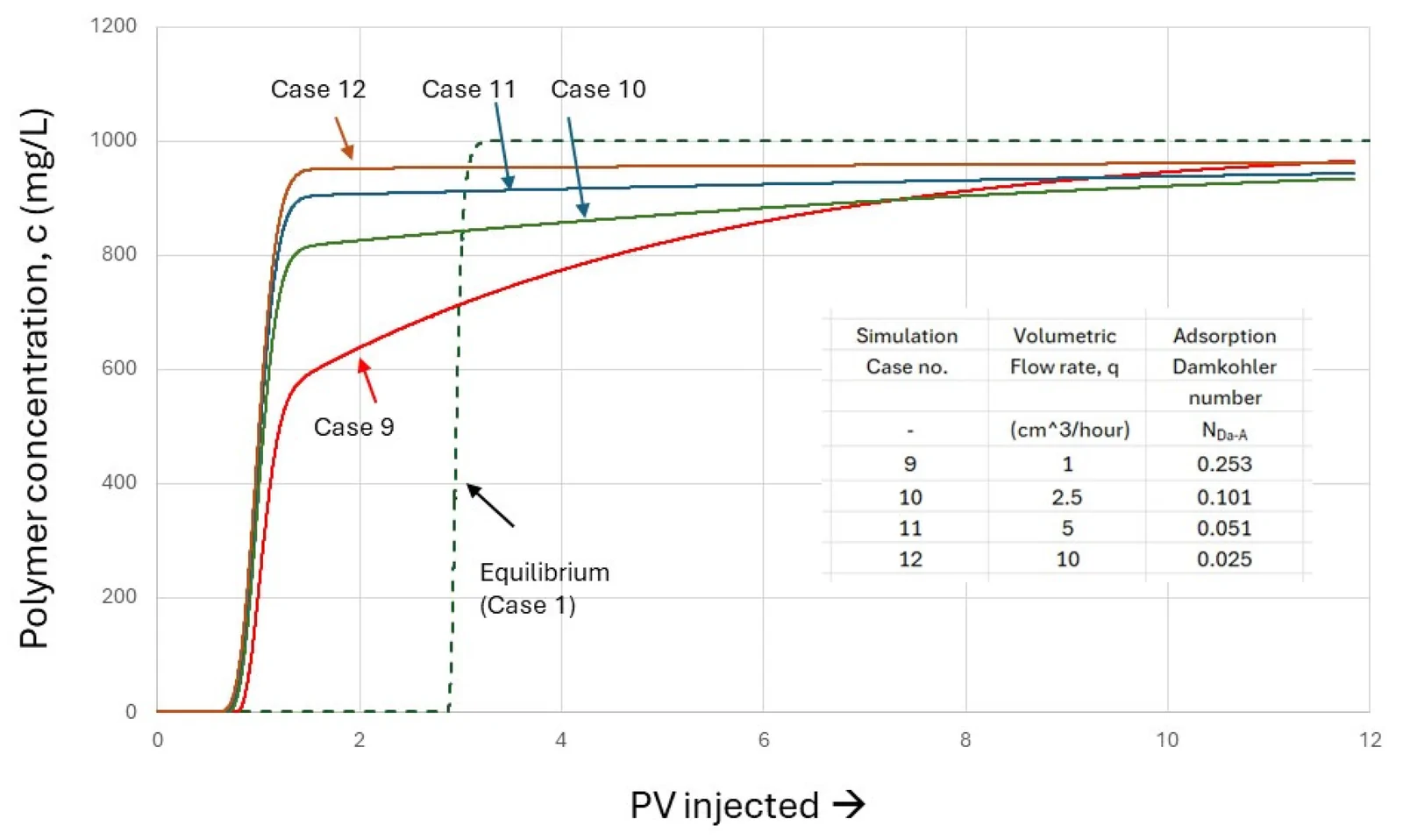
The simulated polymer effluent curve for the “highly non-equilibrium” Cases 9–12, where the effluent profiles are very different from the equilibrium case (Case 1).

*Highly Non-Equilibrium Polymer Effluent Cases (Cases 9–12;* Table 3*):* The highly non-equilibrium polymer adsorption kinetic cases are shown in Figure 14 plotted over the entire flood (0–12 PV). These cases are characterised by showing early breakthrough, which actually closely tracks the tracer breakthrough. This is an important prediction/finding since it may lead the experimentalist to believe that adsorption (often identified by polymer retardation) is very low, when it is in fact not low; it is simply a non-equilibrium effect, and the system has not yet reached its (relatively high) equilibrium adsorption value. A second important prediction, which is best seen in Cases 11 and 12, is that the polymer effluent shows a very long flat plateau-like region *below* the input polymer concentration (c_0_ = 1000 mg/L) for many PV. In fact, it can be seen from the numerical results that the effluent polymer concentration in this “plateau” is actually increasing very slowly (by only a few mg/L per PV injected), and this could not be detected analytically. Experimentally, it may be possible to detect analytically that the effluent is below the input concentration. However, when this has actually been observed in the laboratory, it has sometimes been interpreted as being an error of analysis and the plateau has been re-normalised to the input concentration. Such a “correction” may compound the error in the measurement of polymer adsorption when kinetic effects are present.

**Figure 15:**
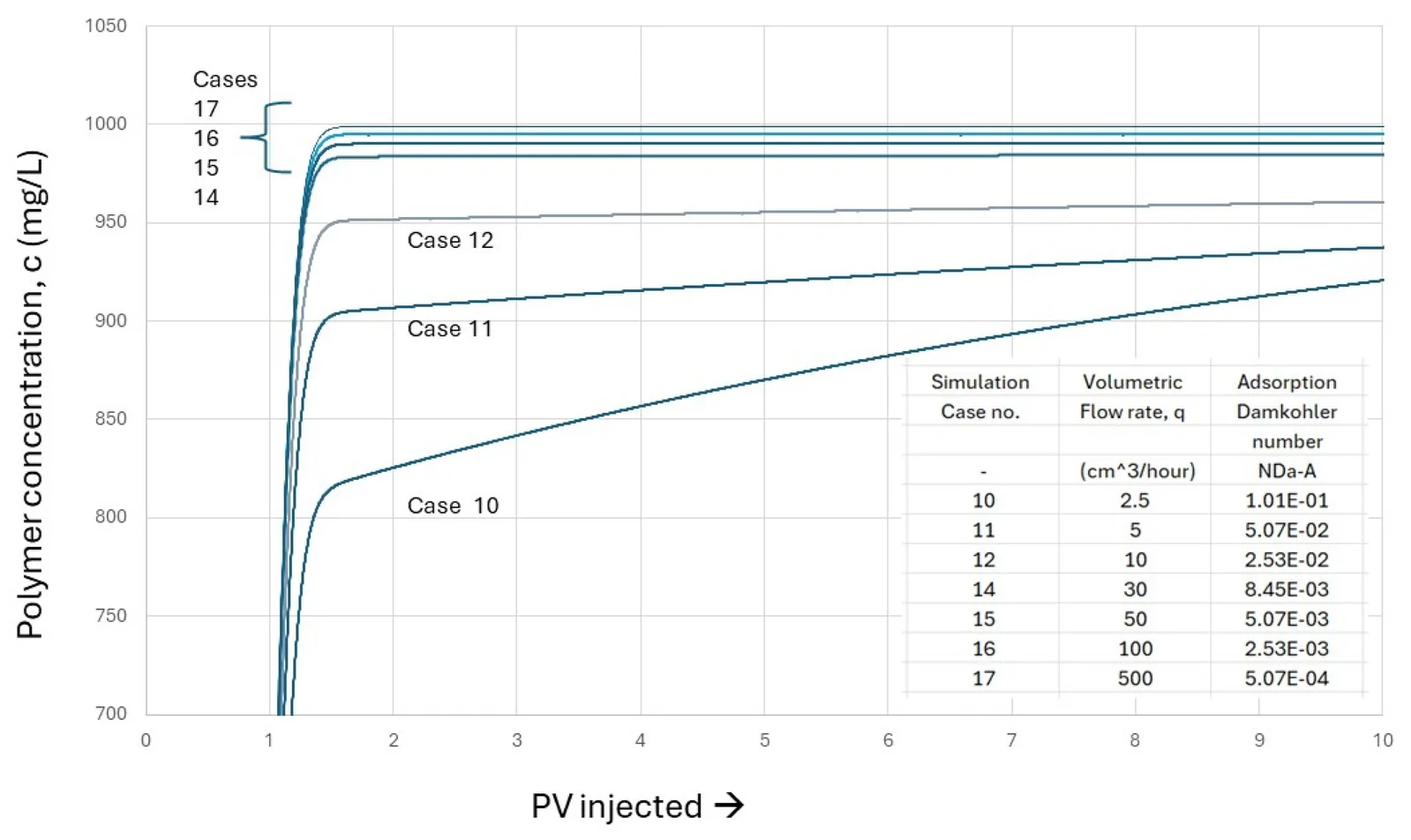
The simulated polymer effluent curves for the “almost tracer-like” Cases 14–17 shown in the context of the “highly non-equilibrium” Cases 10–12. Note that the scale of the polymer effluent is from c = 700 mg/L to 1050 mg/L; the lower breakthrough parts of the effluent are almost merged, as indicated in Figure 11.

**Figure 16:**
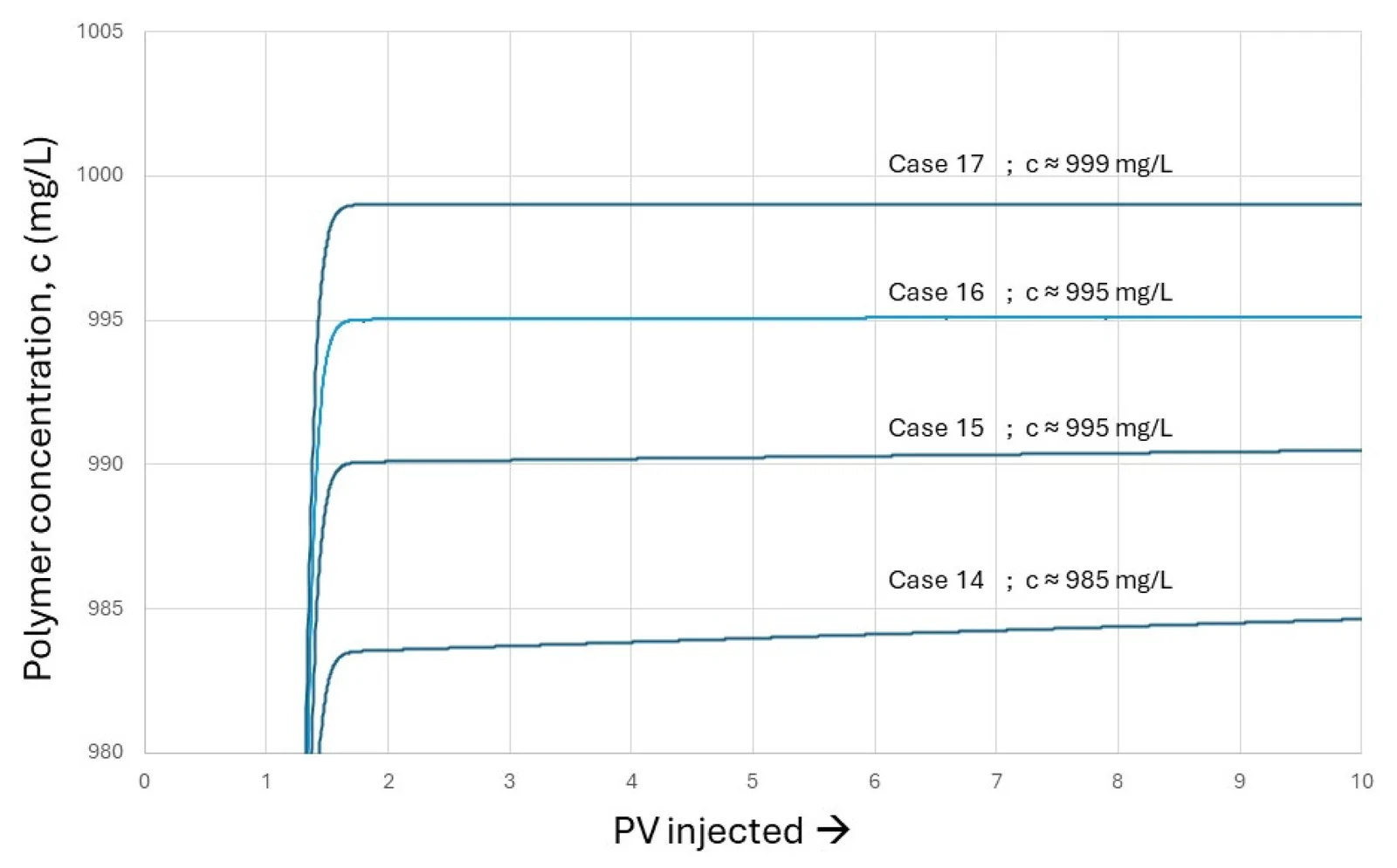
The simulated high part of concentration polymer effluent curves for the “almost tracer-like” Cases 14–17. Note that the scale of the polymer effluent is from c = 980 mg/L to 1005 mg/L; the lower breakthrough parts of the effluent are almost merged, as indicated in Figure 14 and Figure 15.

*Highly Non-Equilibrium Almost Tracer-Like Polymer Effluent Cases (Cases 12–17;* Table 3*):* These highly non-equilibrium almost tracer-like polymer adsorption kinetic cases are shown in Figure 15 plotted over the entire flood (0–10 PV). The scale of polymer concentration in Figure 16 is from c = 700–1050 mg/L, and it can be seen that the group of Cases 14–17 all show plateau behaviour which gradually approaches (but does not quite reach) the input polymer concentration (c_0_ = 1000 mg/L). The lower concentration part of the effluent for these cases almost coincides with the tracer profile. Figure 16 shows a more expanded view of Cases 14–17 for concentrations between 900 and 1005 mg/L, where the actual value of the plateau level at 10 PV of polymer injection is listed on the figure. Although a very clear pattern of behaviour is seen in these calculations, it is doubtful whether current polymer analytical techniques could distinguish a concentration value in this range from the full input concentration (1000 mg/L).

*Overview of the Flow Regimes Due to Non-Equilibrium Effects:* The results in this section show the range of polymer effluent behaviours which can arise due to non-equilibrium adsorption processes. Polymer effluent behaviour has already been reported in the literature, which qualitatively resembles some of the various simulated effluents in this work [15,16,17,18,19].

**Figure 17:**
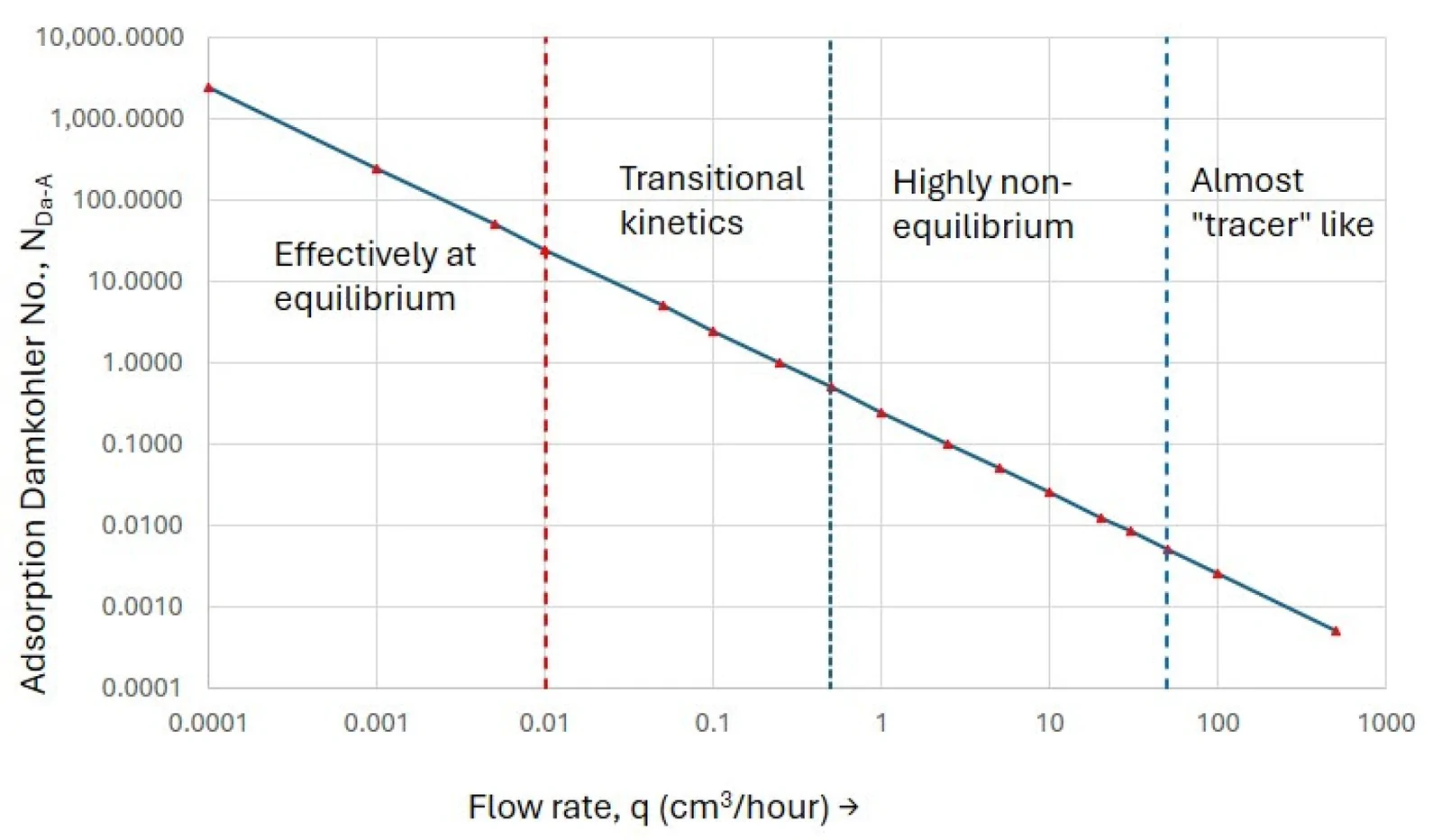
The adsorption Damköhler number, N_Da-A_, as a function of the fluid flow rate, q, (where fluid pore velocity, ν = q/(Aϕ), for the fixed value of adsorption rate constant, k_d_* = 0.01 h^−1^.

The four approximate regimes that can occur are summarised in Figure 17 where the adsorption Damköhler number is shown as a function of volumetric flow rate, q (strictly ν = q/(Aϕ)). The four regimes are described as follows:

○ Near-equilibrium cases with R ≈ N_Ad_ (*∞* > *N_Da_*__*A*_>∼25)
○ Transitional kinetics cases (~5 > *N_Da_*__*A*_>∼0.5)
○ Highly non-equilibrium—with significant “tailing” (~0.25 > *N_Da_*__*A*_>∼0.025)
○ Near tracer-like—showing plateau behaviour with c < c_0_ (~0.013 > *N_Da_*__*A*_ > 0)

### 4.4. Scaling Behaviour of Kinetic Adsorption & Transport

**Figure 18:**
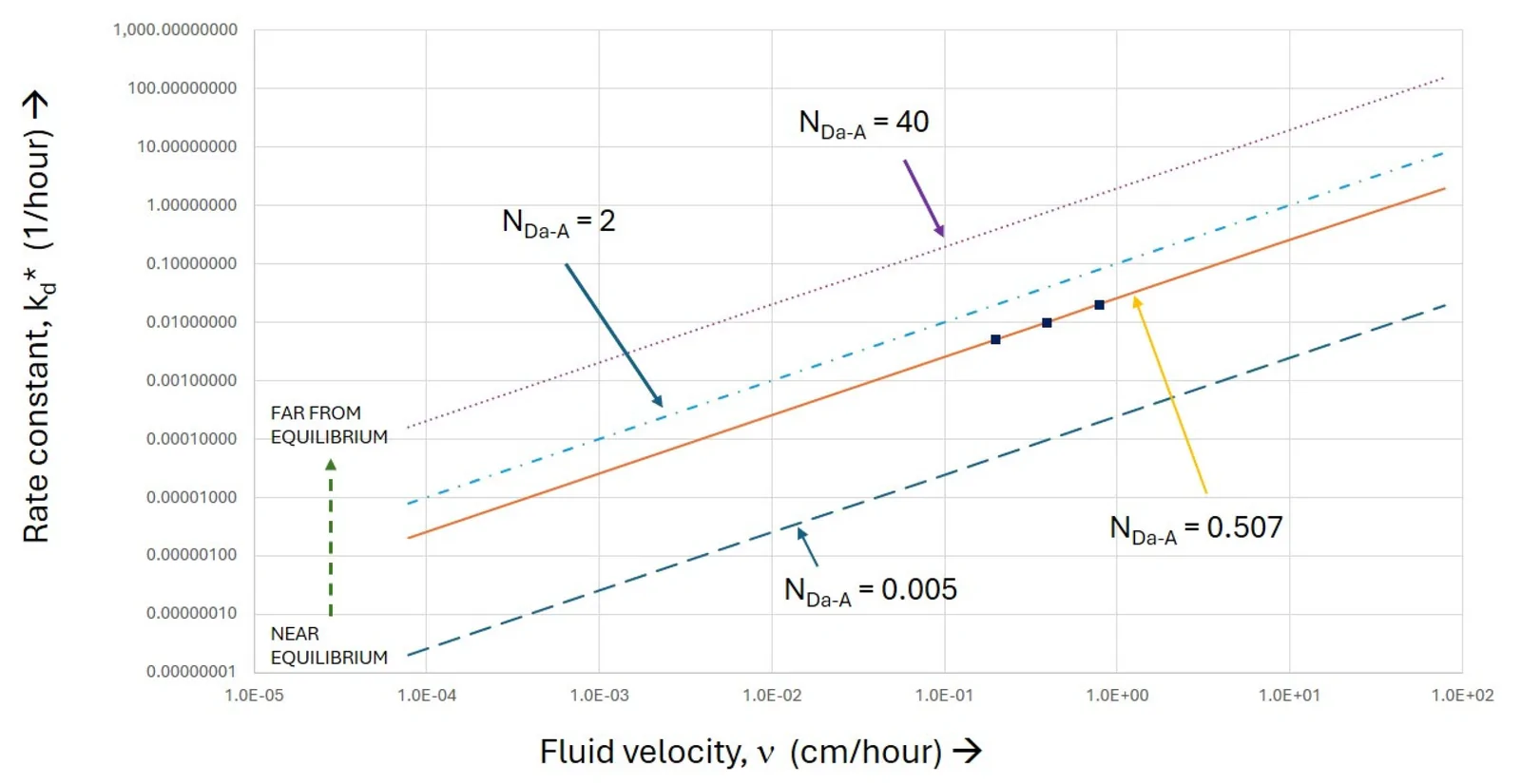
This figure plots the adsorption rate constant (k_d_*) against the fluid velocity (ν), showing the lines of constant N_Da-A_. Combinations of the k_d_*/ν along these lines are exactly scaled and will give identical polymer effluent results; see Figure 19.

The results in Appendix A show that the system is governed by three dimensionless numbers, viz. (i) the Peclet number, N_Pe_, which is of minor importance in these calculations; (ii) the Adsorption number, N_Ad_, which is essentially the equilibrium retardation factor (R), as shown in Figure 10 and (iii) the Adsorption Damköhler number, N_Da-A_, which is a measure of how close or far from equilibrium adsorption the system is located. Thus, for the same Peclet and Adsorption numbers—i.e., the same adsorption isotherm and core properties—systems with the same adsorption Damköhler number are exactly similar. Since, $N_{\mathrm{Da}\_A}=\left(\frac{Lk_{d}^{*}}{v}\right)$, then if the ratio $k_{d}^{*}/\nu$ is constant between any two cases, these two simulations should be exactly identical. This allowed us to take just one k_d_* above and vary the flow rate to span *all* the possibilities from equilibrium through kinetic to almost no adsorption (i.e., all N_Da-A_), as has already been carried out in Section 4.1, Section 4.2 and Section 4.3 above. This leads to Figure 18 which plots the rate constant, k_d_* vs. fluid velocity, ν, showing lines of constant N_Da-A_ = 0.005 (close to equilibrium), 0.507, 2 and 40 (far from equilibrium). Systems having the k_d_*/ν values on any of these constant N_Da-A_ lines are essentially identical, and to illustrate this, calculations for the three points (denoted ■) on the N_Da-A_ = 0.507 line are shown in 8.

**Figure 19:**
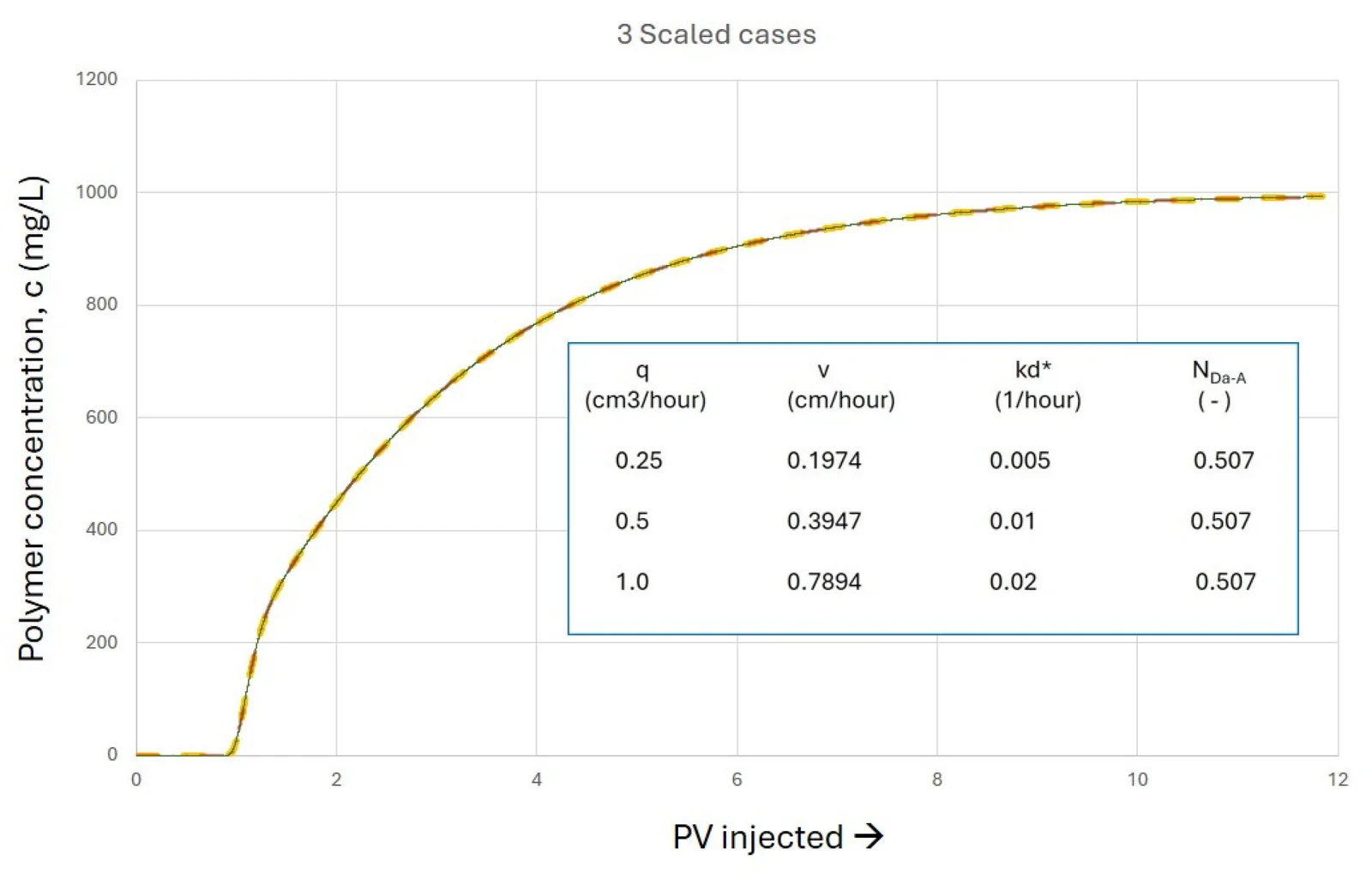
A plot of the effluent profiles from three exactly scaled simulations with N_Da-A_ = 0.507; these are shown as the three points (■) on this line in Figure 18.

Figure 19 shows the simulated effluent for Case 8 (q = 0.4 cm^3^/hour) in Table 3 (for which N_Da-A_ = 0.507)—the other two scaled cases are taken by halving and doubling both the ν and k_d_* values such that the N_Da-A_ value is preserved. Clearly, these effluents are identical (to ~6 decimal places), thus demonstrating the exact scaling of these systems.

### 4.5. The Effect of “Shut-Ins” on the Polymer Effluents

The clear hypothesis from the simulations presented above in Section 4.3 is that the form of the polymer effluent profile is strongly affected by how close to, or far from, equilibrium the system is (the value of N_Da-A_). This work implies that the strong “tailing” observed in polymer core adsorption experiments is due to non-equilibrium effects. This may or may not be the case, but the central question is: what predictions can be made—or how should these polymer displacement experiments be performed—such that we can confirm or refute this hypothesis?

For example, it was noted above that in the case of non-equilibrium behaviour where plateau levels below the input value were observed (e.g., Cases 11–17) or where there is considerable tailing, then it may not be certain that this is due to kinetic effects on the adsorption. In fact, the role of the kinetics can be shown by predicting what would happen when we stop the flow and then restart; i.e., we perform a “shut-in” in the core flooding sequence. Clearly, if the system is not at equilibrium, then on a shut-in, it will re-equilibrate to some extent, and there will be some resultant change in the effluent profiles. This would not happen if the entire flow and adsorption process were at full adsorption equilibrium; in this latter case, nothing should be observed, and the effluent should continue to rise with no break or discontinuity.

To test this theoretically, we performed the core flood simulations including such a shut-in period. Injection of the polymer is simulated for a given period of time until sometime after breakthrough at the outlet, and then the flow is stopped (ν = 0) for a period of time. During this shut-in time, according to the governing equations (Equations (11) and (12)), no transport occurs, but the adsorption kinetics (Equation (12)) continues and, in the long term, would reach local equilibrium.

**Figure 20:**
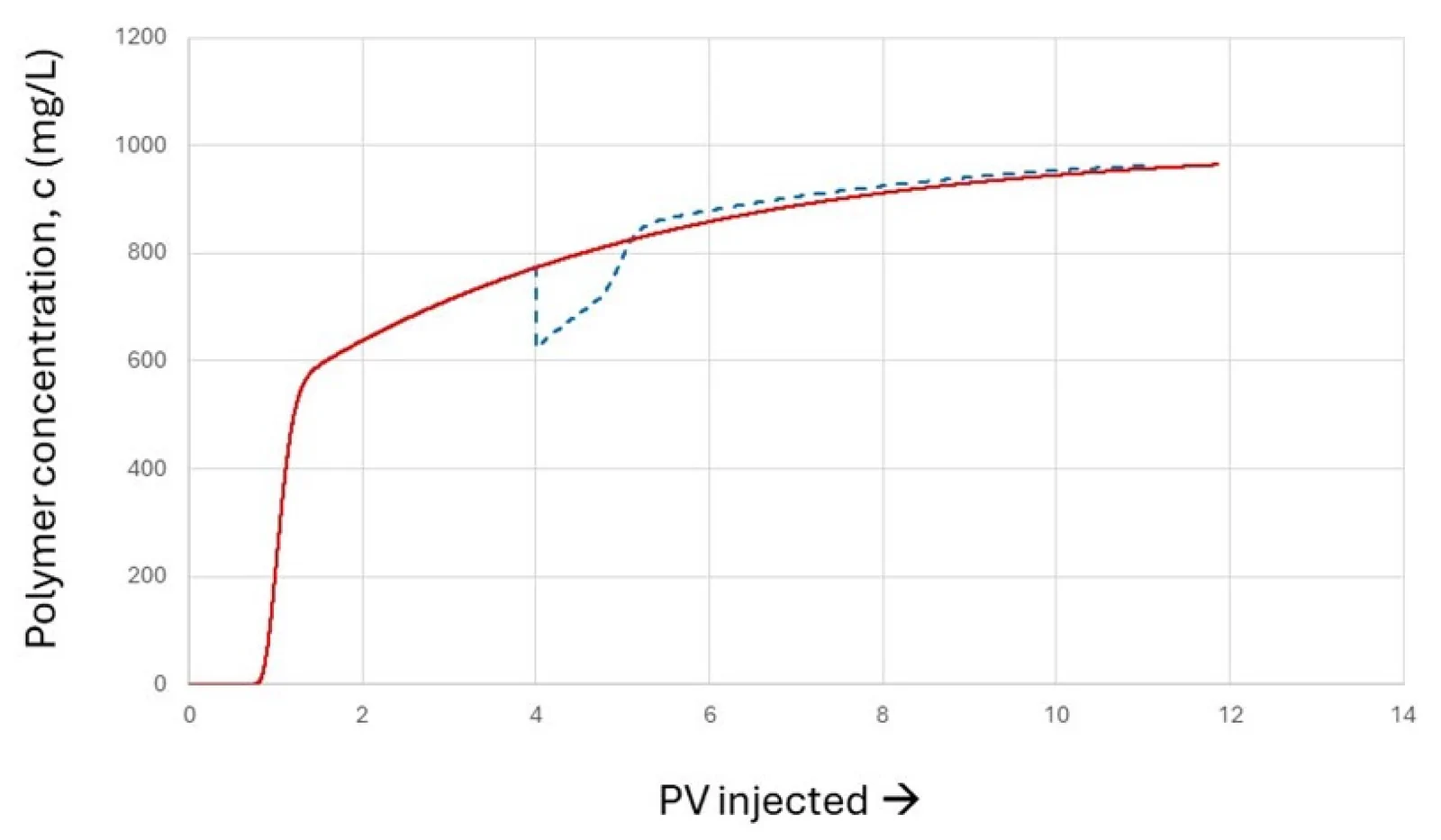
Case 9 (q = 1 cm^3^/h) showing 12 PV of continuous polymer injection compared with the predicted polymer effluent profile with a 40 h shut-in after 4 PV of injection.

**Figure 21:**
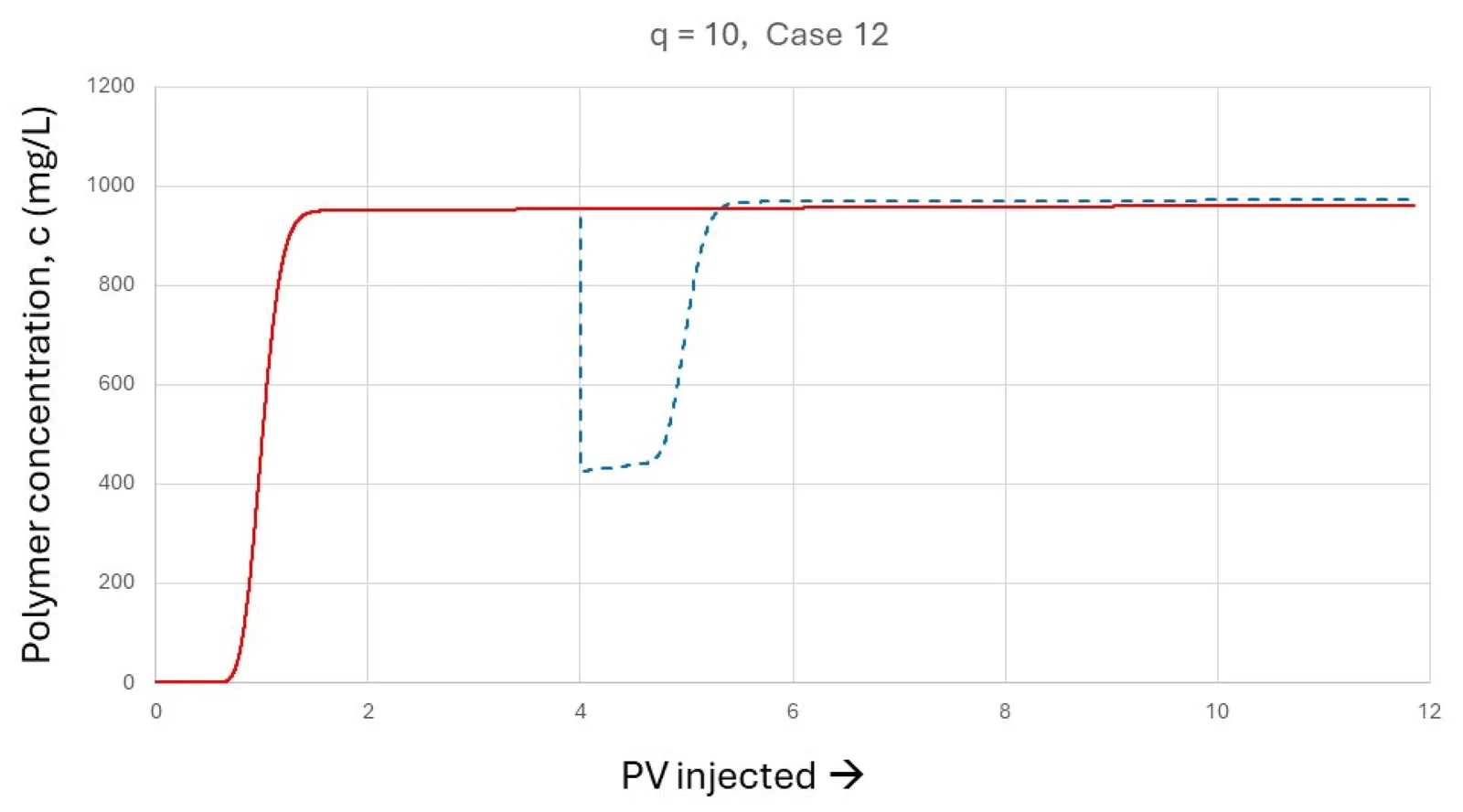
Case 12 (q = 10 cm^3^/h) showing 12 PV of continuous polymer injection compared with the predicted polymer effluent profile with a 40-h shut-in after 4 PV of injection.

The results from two simulations of this type are presented in Figure 20 and Figure 21 based on Cases 9 and 12 in Table 3. In each case, 4 PV of polymer (again at c_o_ = 1000 mg/L) was injected, followed by a 40-h shut-in period, after which the flow is resumed at the same flow rate, and results in both cases are compared with the original polymer effluent profiles with continuous injection (no shut-in). In both cases, a very clear drop in the effluent polymer concentration is observed, and as may be expected, the drop is largest in the Case 12 example since this is furthest from equilibrium (i.e., it has the larger N_Da-A_). It is also discernible in both Figure 20 and Figure 21 that the dip in effluent polymer concentration in both cases lasts for approximately 1 PV and is of a magnitude which should be easily detectable experimentally. After this dip, the polymer concentration rises to a level just above the continuous injection case. Again, this may be expected qualitatively, but the magnitude of the polymer concentration increase following the shut-in would be difficult to detect experimentally using conventional polymer assay techniques.

**Figure 22:**
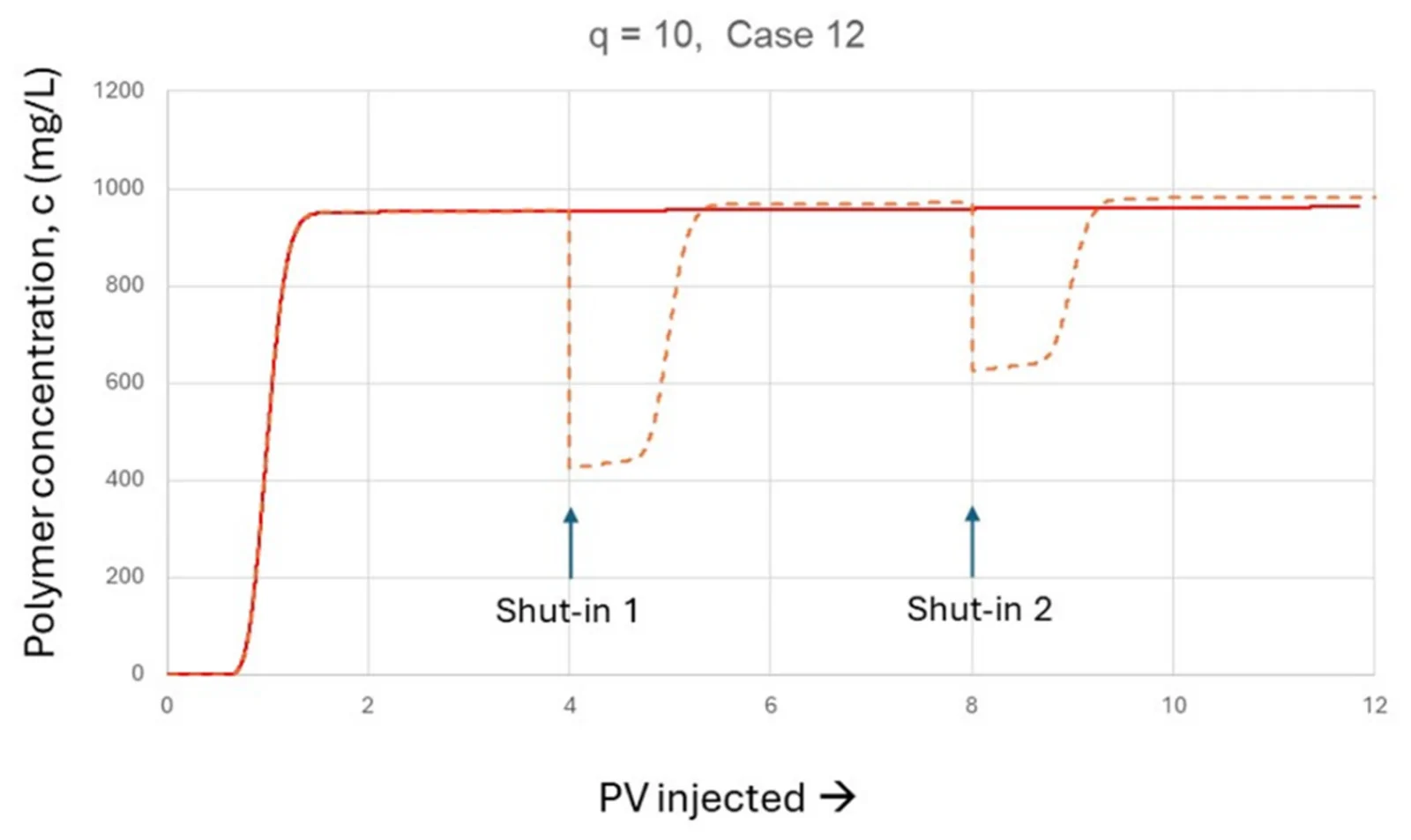
Case 12 (q = 10 cm^3^/h) showing the continuous polymer injection compared with the predicted effluent profile with a 40-h shut-in after 4 PV of injection, and then a second 40-h shut-in after 8 PV of injection.

Experimentally, there is no particular difficulty in carrying out multiple shut-in stages in a polymer core or mineral pack flood. The effect of *two* shut-ins is shown for Case 10 in Figure 22. In this case, both shut-in stages show a clear response in the polymer effluent dip since the system is quite far from equilibrium, although this will depend on the system.

### 4.6. The Effect of the Shape of the Equilibrium Adsorption Isotherm

The results presented in this paper so far have been based on the example of the Freundlich equilibrium adsorption isotherm represented by Equation (2) and shown in Figure 9a; which also appears as Γ_1_(c) in Figure 9b. Any other polymer adsorption isotherm of this type (i.e., concave downwards, monotonic with a plateau region), such as the Langmuir isotherm, would obviously give the same value of Adsorption number, N_Ad_, if it had the same Γ_max_ at c_o_. In addition, it would be expected to behave in a qualitatively similar manner, and this has been checked for the Langmuir isotherm (results not presented here). However, the question arises: does the *shape* of the equilibrium isotherm have any unexpected interaction with the kinetics, as described in this work? In particular, is there an observable effect if the isotherm is a very steeply rising “stripping” isotherm as shown for the 2 cases, Γ_2_(c) and Γ_3_(c), in Figure 9b? The formulae for the three cases are shown inset on Figure 9b.

**Figure 23:**
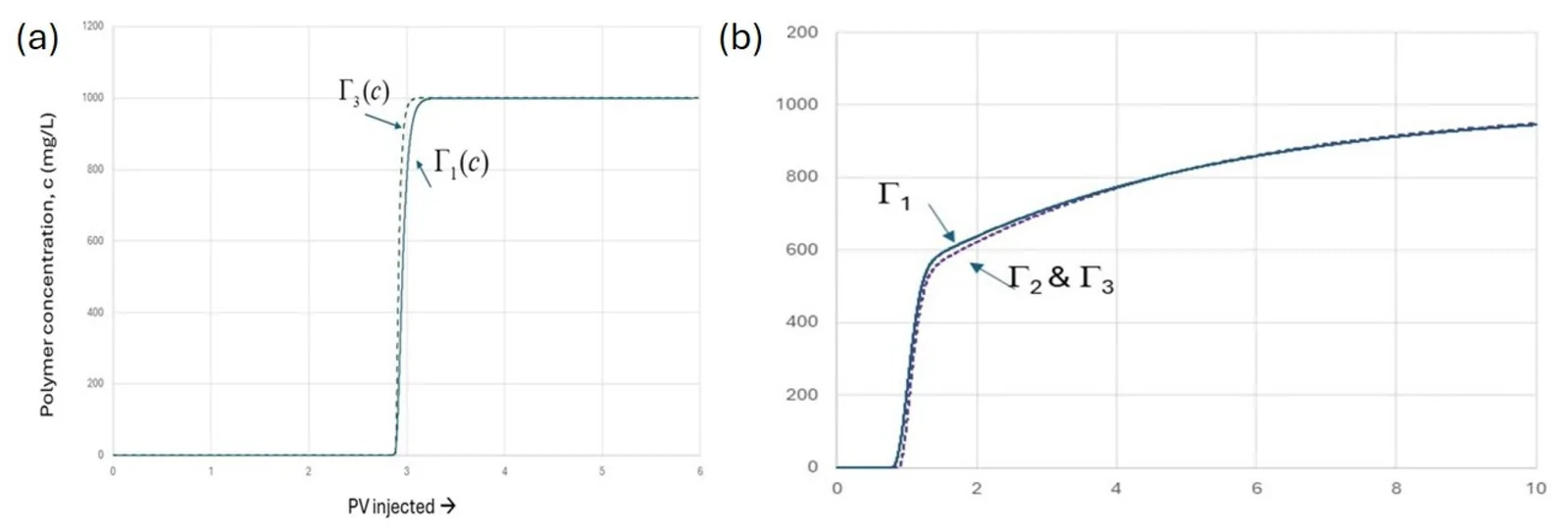
A comparison between the effluent profiles for the three adsorption isotherms shown in Figure 9b for (**a**) the equilibrium case and (**b**) for the non-equilibrium Case 9 (q = 1 cm3/h). The base-case isotherm (Table 2) is Γ_1_ (β = 0.25) and the two “stripping” isotherms are Γ_2_ (β = 0.04) and Γ_3_ (β = 0.01).

From the analysis of the dimensionless flow equations, Equations (A12) and (A13) (Appendix A), it can be seen that the three dimensionless parameters (*N_Pe_*, *N_Ad_* and *N_Da_*__*A*_) are all the same, but the Freundlich exponent β differs between the 3 cases (Equation (A13)); β = 0.25 (base case, Γ_1_), 0.04 (Γ_2_) and 0.01 (Γ_3_). The latter case (0.01) is close to an assumption that polymer retention/adsorption is almost independent of polymer concentration, which has been a common assumption among some porous media researchers [18]. A direct comparison between the effluent profiles for the three forms of the adsorption isotherm is shown in Figure 23a for the equilibrium case and in Figure 23b for the non-equilibrium Case 9 (q = 1 cm^3^/h). The far-from-equilibrium Case 12 (q = 10 cm^3^/h) was also simulated, but the three isotherm cases gave virtually identical results. The equilibrium cases in Figure 23a show that the stripping isotherm (Γ_3_) is slightly sharper than the base case (Γ_1_), and the other case (Γ_2_) lies very close to the Γ_3_ case. Figure 23b shows the greatest deviation of the two stripping isotherms (Γ_2_ and Γ_3_) from the base case (Γ_1_) for the range of non-equilibrium cases simulated. This demonstrates fairly conclusively that the range of effluent behaviours observed is principally due to the effect of the adsorption kinetics, and it has little dependence on the precise form of the equilibrium adsorption isotherm.

## 5. Summary and Conclusions

In this paper, a model for the transport and kinetic adsorption of polymer flowing through porous media is developed and applied to both (i) understand some of the observed behaviour of the polymer effluent profiles from core floods, and (ii) to make some testable experimental predictions. It is assumed that the underlying equilibrium adsorption isotherm, Γ(c), is of Freundlich form, and hence the kinetic expressions used to describe polymer adsorption and desorption are consistent with this form. However, it is shown that the precise form of the adsorption isotherm is not of major importance and any equilibrium form, such as the Langmuir or Sips equations, leads to virtually identical conclusions. The main issue is that the adsorption isotherm is monotonic, concave downwards, shows steeply rising behaviour at low concentration and has a fixed maximum adsorption level at the injected polymer concentration, c_0_, or has a plateau in the adsorption at higher polymer concentrations (not essential).

The coupled set of two equations (Equations (15) and (16)) for polymer transport and the kinetic adsorption of polymer governs the transport and kinetic adsorption process. The dimensionless form of these equations (Equations (17) and (18)) show that the system behaviour entirely depends on three dimensionless numbers, viz. the Peclet number, *N_Pe_*, the Adsorption number, *N_Ad_*, and the adsorption Damköhler number, *N_Da_*__*A*_. Only the latter two are of major importance in this work. The Adsorption number, *N_Ad_*, is identical to the quantity R (R = *N_Ad_*) which is the amount that polymer effluent is retarded behind the tracer in an *equilibrium* adsorption system. The adsorption Damköhler number, $N_{\mathrm{Da}\_A}=\left(Lk_{d}^{*}/\nu \right)$, describes the ratio of the fluid residence time in the system, (L/ν) to the timescale of the adsorption process (1/k_d_*). As *N_Da_*__*A*_ → *∞*, the system approaches equilibrium; that is where the adsorption level on the porous medium and in the fluid locally instantaneously obey the equilibrium adsorption isotherm. As *N_Da_*__*A*_ → 0, the system tends toward non-adsorbing “tracer-like” behaviour, in that the polymer breakthrough curve is almost the same as the non-adsorbing tracer effluent curve.

The main conclusions of this work are as follows:

(i) When simulating a displacement in a polymer adsorption core flood, the polymer transport and kinetic adsorption model shows a wide range of polymer effluent regimes, which can be broadly classified as:
  ○ Near-equilibrium cases with R ≈ N_Ad_ (*∞* > *N_Da_*__*A*_>∼25)
  ○ Transitional kinetics cases (~5 > *N_Da_*__*A*_>∼0.5)
  ○ Highly non-equilibrium—with significant “tailing” (~0.25 > *N_Da_*__*A*_>∼0.025)
  ○ Near tracer-like—showing plateau behaviour with c < c_0_ (~0.013 > *N_Da_*__*A*_ > 0)
(ii) From the fact that the three dimensionless groups listed above control the entire transport and kinetic polymer adsorption process, it is predicted that identical results can be achieved *either* by (a) taking a fixed kinetic rate (k_d_*—and hence k_a_*) and varying the flow rate, i.e., q or ν= q/(Aϕ), *or* (b) by fixing the injection rate and varying the rate of adsorption (i.e., k_d_*—and k_a_*) such that N_Da-A_ is preserved. This scaling is represented in Figure 18, and example calculations are given to confirm this exact scaling (Figure 19).
(iii) The model polymer effluent predictions are qualitatively similar to experimental core flood results appearing in the literature; this is evident for the experimental results in Figure 2 (Seright and Wang, [19]) and in related publications by Silva et al. [15,16]. A direct simulation of these data to match some of these results is currently in progress.
(iv) If the kinetic adsorption effect is the correct explanation of the “tailing” and “plateau” behaviour in the polymer effluent profiles in experimental core floods, then we demonstrate how this can be established. It is shown that by performing a “shut-in” (i.e., reducing the injection rate to q = 0) for a period of some hours (~ 5 to 40 h), depending on the timescale of the adsorption process, then a dip in polymer concentration will be observed in the measured effluent. Preliminary polymer adsorption core flood experiments have confirmed this finding [16].
(v) The shape of the polymer adsorption isotherm (see Figure 9) was radically changed from the gradually rising case Freundlich function (Γ_1_) to successively sharper “stripping” isotherms (Γ_2_ and Γ_3_); R = *N_Ad_* was identical for all 3 cases. This had a very small effect on the equilibrium breakthrough polymer effluent profiles, where the stripping cases gave a slightly sharper breakthrough profile for the more stripping isotherm cases (Γ_2_ and Γ_3_). However, this had a *negligible* effect on the polymer effluent profiles in the non-equilibrium region, and the corresponding cases had almost identical profiles for Γ_1_ and Γ_2_ /Γ_3_. This demonstrates that the exact form of the equilibrium adsorption isotherm has little effect on the non-equilibrium polymer effluent profiles.

In future work, we intend to apply the above principles to test whether they are sufficient to explain the dynamic retention experiments of Seright and Wang [18,19] and other observations in the literature [15,16]. Preliminary results are optimistic and will be presented in a forthcoming paper.

## Figures and Tables

**Table 1:** Example data for the kinetic Freundlich model described by Equations (9) and (10).

Property	Symbol	Value	Unit
Mass	m	20	g
Volume	V	0.15	L
Polymer initial concentration	c_0_	100	mg/L
Rate constant, adsorption	k_a_*	0.05	(mg/L)^1−β^/h
Rate constant, desorption	k_d_*	0.01	1/h
Freundlich exponent,	β	0.25	Dimensionless
Calculated, Freundlich constant α = (V/m)(k_a_*/k_d_*)	α	0.0375	mg^(1−β)^L^β^g^−1^

**Table 2:** Base case example data for the polymer transport kinetic Freundlich model.

1D Model Core		1D Grid		Polymer Data	
Input					
Core length, L =	20 cm	Number of grid blocks, NX=	40	Adsorption rate constant k_a_* =	3.5 (mg/L)^1−β^/h
Porosity, ϕ =	0.25			Desorption rate constant, k_d_* =	0.01 1/h
Area, A =	5.0671 cm^2^			Freundlich beta β =	0.25
Rock density, ρ=	2.64 g/cm^3^			Concentration, c_0_=	1000 mg/L (ppm)
**Calculated**					
Pore volume =	25.34 cm^3^ 2.534 ×10^−2^ L	Volume of 1 grid block, V	6.334 × 10^−4^ L	Freundlich alpha $\alpha =\left(\frac{V.k_{a}^{*}}{m.k_{d}^{*}}\right)$	0.03314 mg^(1−β)^L^β^g^−1^
		Mass 1 grid block, m	6.689 g	Maximum adsorption *Γ_max_ = Γ(*c_0_*)*	0.18636 mg/g 186.36 μg/g
		Grid size, Δx	0.5 cm	Retardation factor, R, or Adsorption number, N_Ad_, where R = N_Ad_	1.968
		Peclet number *N_Pe_* = 2L/Δx	80		

**Table 3:** Polymer transport and kinetic adsorption simulations for the continuous polymer injection cases carried out in this study.

Simulation Case No.	Volumetric Flow Rate, q	Adsorption Damkohler Number	Residence Time in Core (Hour)	Number of Half-Lives, t1/2	Fluid Pore Velocity v = q/(Af)	Adsorption Type
-	(cm^3^/hour)	N_Da-A_	(Hour)	-	(cm/hour)	
1	0.0001	2.534E+03	253,353.7	3655.1	7.894E-05	Near Equilibrium
2	0.001	2.534E+02	25,335.4	365.5	7.894E-04	
3	0.005	5.067E+01	5067.1	73.1	3.947E-03	
4	0.01	2.534E+01	2533.5	36.5	7.894E-03	
5	0.05	5.070E+00	506.7	7.3	3.947E-02	Transitional Kinetic
6	0.1	2.530E+00	253.4	3.7	7.894E-02	
7	0.25	1.010E+00	101.3	1.5	1.974E-01	
8	0.5	5.070E-01	50.7	0.731	3.947E-01	
9	1	2.530E-01	25.3	0.366	7.894E-01	Highly Non-equilibrium
10	2.5	1.013E-01	10.1	0.1462	1.974E+00	
11	5	5.067E-02	5.067	0.0731	3.947E+00	
12	10	2.534E-02	2.534	0.0366	7.894E+00	
13	20	1.267E-02	1.267	0.0183	1.579E+01	Almost “Tracer like”
14	30	8.445E-03	0.845	0.0122	2.368E+01	
15	50	5.067E-03	0.507	0.0073	3.947E+01	
16	100	2.534E-03	0.253	0.0037	7.894E+01	
17	500	5.067E-04	0.051	0.00073	3.947E+02	

## Data Availability

The original contributions and data presented in this study are included in the article. Further inquiries can be directed to the corresponding author.

## Appendix A. The Dimensionless Form of the Polymer Transport and Kinetic Adsorption Equations

It is important to consider the dimensionless form of the polymer transport and kinetic adsorption equations (Equations (15) and (16)) since this brings out the scaling behaviour of the kinetic system. The transport/adsorption groups apply when the fluid transport velocity *v* ≠ 0. The dimensionless concentration (C), distance (X) and time (T) are defined as follows:

(A1) $$C=\frac{c}{c_{o}};X=\frac{x}{L};T=\frac{v.t}{L}$$

and the dimensionless adsorption ($\tilde{\Gamma }$) is defined as follows:

(A2) $$\tilde{\Gamma }=\frac{\Gamma }{\Gamma (c_{o})}=\frac{\Gamma }{\Gamma_{\max}}$$

Applying these first to the transport equation (Equation (15)) gives:

(A3) $$\left(\frac{\partial C}{\partial T}\right)=\frac{D}{\mathrm{vL}}\left(\frac{\partial^{2}C}{\partial X^{2}}\right)-\left(\frac{\partial C}{\partial X}\right)-\left(\frac{\rho \Gamma_{\max}}{\phi c_{o}}\right)\left(\frac{\partial \tilde{\Gamma }}{\partial T}\right)$$

where the two dimensionless groups which emerge are the Peclet number (*N_Pe_*) and the adsorption number (*N_Ad_*) which are defined as follows:

(A4) $$N_{\mathrm{Pe}}=\frac{\mathrm{vL}}{D}\;\mathrm{and}\;N_{\mathrm{Ad}}=\left(\frac{\rho \Gamma_{\max}}{\phi c_{o}}\right)$$

And hence Equation (A3) is expressed in fully dimensionless form as:

(A5) $$\left(\frac{\partial C}{\partial T}\right)=\frac{1}{N_{\mathrm{Pe}}}\left(\frac{\partial^{2}C}{\partial X^{2}}\right)-\left(\frac{\partial C}{\partial X}\right)-N_{\mathrm{Ad}}\left(\frac{\partial \tilde{\Gamma }}{\partial T}\right)$$

The Peclet number plays a very minor role in the central subject of this paper (kinetics of polymer adsorption in transporting systems) as the flows are broadly convection dominated, with typically high values of *N_Pe_*≥~80 However, the Adsorption number, *N_Ad_*, is very important since it expresses the magnitude of the retardation factor, *R*, of the adsorbing polymer. An inert tracer transported through a 1D core or porous pack will break through just before 1 PV of injection and will have a characteristic sigmoidal breakthrough with a C = c/c_0_ = 0.5 at 1 PV. However, for an adsorbing species in ***equilibrium*** adsorption, it will be retarded, and *R* is the number of pore volumes beyond 1 PV where C = 0.5 occurs in the effluent curve. The Adsorption number can be expressed in different equivalent ways and, in polymer core and pack flooding, the following equivalence is straightforward to show:

(A6) $$R=\left(\frac{M_{\mathrm{core}}.\Gamma (C_{o})}{V_{p}.C_{o}}\right)=N_{\mathrm{Ad}}=\left(\frac{\rho \Gamma_{\max}}{\phi c_{o}}\right)$$

Now consider the conversion of Equation (16) describing the coupled kinetic polymer adsorption, which only requires the dimensionless values of C, T and $\tilde{\Gamma }$ above (not X). Transforming Equation (16) with these dimensionless variables gives Equation (A7):

(A7) $$\left(\frac{\partial \tilde{\Gamma }}{\partial T}\right)=\left(\frac{\mathrm{LV}k_{a}^{*}c_{o}^{\beta }}{\mathrm{vm}\Gamma_{\max}}\right)C^{\beta }-\left(\frac{Lk_{d}^{*}}{v}\right)\tilde{\Gamma }$$

From Equation (5) for α in the main text of this paper, then $\alpha k_{d}^{*}=\left(\frac{Vk_{a}^{*}}{m}\right)$, and this gives:

(A8) $$\left(\frac{\partial \tilde{\Gamma }}{\partial T}\right)=\left(\frac{\mathrm{L\alpha}k_{d}^{*}c_{o}^{\beta }}{v\Gamma_{\max}}\right)C^{\beta }-\left(\frac{Lk_{d}^{*}}{v}\right)\tilde{\Gamma }$$

which simplifies to:

(A9) $$\left(\frac{\partial \tilde{\Gamma }}{\partial T}\right)=\left(\frac{Lk_{d}^{*}}{v}\right)\left[\left(\frac{\alpha c_{o}^{\beta }}{\Gamma_{\max}}\right)C^{\beta }-\tilde{\Gamma }\right]$$

And the two dimensionless groups which emerge are as follows:

(A10) $$(i)\;\mathrm{The}\;\mathrm{adsorption}\;\mathrm{Damköhler}\text{-}\mathrm{type}\;\mathrm{number},\;N_{\mathrm{Da}\_A}=\left(\frac{Lk_{d}^{*}}{v}\right)$$

(A11) $$(\mathrm{ii})\;A\;\mathrm{relative}\;\mathrm{adsorption}\;\mathrm{group}=\left(\frac{\alpha c_{o}^{\beta }}{\Gamma_{\max}}\right)$$

The Damköhler number, *N_Da_*__*A*_, is clearly the ratio of the residence time of the polymer in the core, (*L*/*v*), to the timescale of the adsorption process, $\left(1/k_{d}^{*}\right)$. This will be very important in the calculations of kinetic adsorption, and it also makes an important scaling prediction. *N_Da_*__*A*_ shows that if the single rate constant for the adsorption process, $k_{d}^{*}$, is doubled, then doubling the fluid velocity gives an identical value of *N_Da_*__*A*_ and hence the system is exactly the same. The important implication for this work is that, to study the effects of polymer adsorption kinetics in transport, we can either (i) systematically vary the rate constant $k_{d}^{*}$(remembering to change $k_{a}^{*}$ by the same factor and keeping ν constant), or (ii) keeping $k_{d}^{*}$ (and $k_{a}^{*}$) constant and varying the velocity, ν.

Finally, the relative adsorption group in Equation (A11) is quite clearly = 1, since $\alpha c_{o}^{\beta }$ is identical to Γ_max_. And hence the complete set of dimensionless equations for polymer transport and kinetic adsorption is as follows:

(A12) $$\left(\frac{\partial C}{\partial T}\right)=\frac{1}{N_{\mathrm{Pe}}}\left(\frac{\partial^{2}C}{\partial X^{2}}\right)-\left(\frac{\partial C}{\partial X}\right)-N_{\mathrm{Ad}}\left(\frac{\partial \tilde{\Gamma }}{\partial T}\right)$$

and

(A13) $$\left(\frac{\partial \tilde{\Gamma }}{\partial T}\right)=N_{\mathrm{Da}\_A}\left[C^{\beta }-\tilde{\Gamma }\right]$$

where $0\leq C\leq 1\;\mathrm{and}\;0\leq \tilde{\Gamma }\leq 1$.
